# Stomata biosilica and *Equisetum* photosynthesis: ionic tomography insight using a PDMPO silicaphilic probe†

**DOI:** 10.1039/d4sc07973f

**Published:** 2025-04-24

**Authors:** Victor V. Volkov, Graham J. Hickman, Carole C. Perry

**Affiliations:** a Interdisciplinary Biomedical Research Centre, School of Science and Technology, Nottingham Trent University Clifton Lane Nottingham NG11 8NS UK carole.perry@ntu.ac.uk

## Abstract

Confocal microscopy using silicaphilic molecular probes is a promising approach to identify the ionic character of silica interfaces. Using *ab initio* and density functional theory we model structural and electronic properties of the (2-(4-pyridyl)-5(4-(2-dimethyl-aminoethyl-aminocarbamoyl)-methoxy)phenyl)-oxazole (PDMPO) chromophore at different protonation states, in vacuum, and when next to silica of different ionicity. For protonated chromophores next to anionic silica sites, theory suggests strong emission in the visible spectral range from higher excited states and the probability of weaker near infrared fluorescence from a lower energy manifold. Using theory insights, we conduct single- and two-color confocal microscopy in the visible and in the near infrared, respectively, to study open and closed stomata of *Equisetum arvense*, a heavily silicified primitive plant. Three-dimensional ionic tomography resolves sub-micron neighbouring regions of high and low ionic charges of *exo*/*endo*-skeletal silica components according to whether they are open or closed. Considering the variance of methane and carbon dioxide levels prior to, during and after the Silurian, we discuss the observed high ionic contrast of stomatal apertures upon opening as a signature of bioinorganic machinery able to moderate methane and carbon dioxide transport for optimal growth under a range of atmospheric conditions.

## Abstract 2

Confocal microscopy using silicaphilic molecular probes is a promising approach to identify the ionic character of silica interfaces.

## Graphical abstract

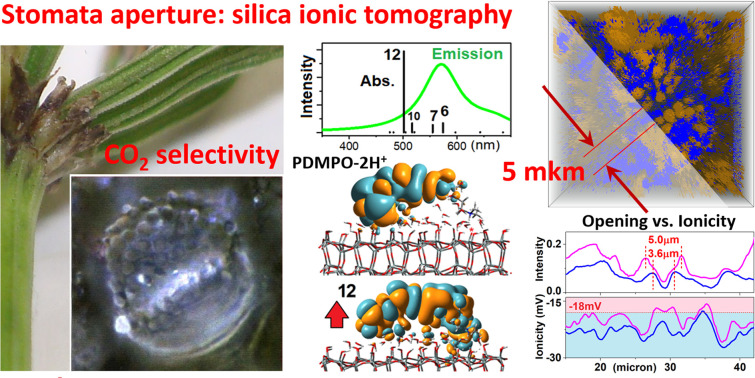

## Introduction

In contrast to calcium-based skeletal multicellular life, there are other organisms which utilise silica, such as some protozoa,^1,2^ algae^3^ including diatoms,^4^ sponges,^5^ as well as higher plant species^6^ for structure, protection and chemical attuning of the local environment. Biosilicification thus provides inspiration for materials engineering. Indeed, silica materials have provided numerous initiatives in biosensor engineering,^7,8^ drug delivery,^9–11^ biomedical technologies,^12,13^ and platforms for cellular growth.^14^

Biosilica is inspirational where its structural strength and mobility are combined. In a previous study we addressed such a case, describing the properties of *Equisetum* spore elators, which facilitate the spread of spores prior to germination.^15^*Equisetum* size-adjustable pores (stomata) are another such example. Stomata evolved around the Silurian to allow the common plant ancestor conquer the land.^16^ Stomata allow/facilitate diffusion of CO_2_ and water.^17^*Equisetum* stomata have been reported to be sensitive (closed-open) to light, CO_2_ and humidity,^18,19^ as well as to the hormone abscisic acid,^20^ according to growing season and species.^21^ While obviously peculiar, the structural properties of stomata attracted research attention. Early studies using light microscopy and scanning electron microscopy provided us with a general outline of their organization and composition.^22–26^ A horsetail stomatal complex consists of two chloroplast-free guard cells (below or inside), and two subsidiary cells (above or outside). The latter shape the pore slit (the margins of which are bordered by pronounced papilla-like teeth), and are silicified upon maturation.^22,23^

Electron microprobe analysis data presented as counts per second (cps) of silicon gave an understanding of the variation of ‘silicon’ deposition (teeth along the margins of the stomatal apertures (193 cps), silica knobs on the surfaces of both the subsidiary cells (105 cps), silica knobs at the edges of the subsidiary cells distal to the aperture (363 cps) and in the wall of subsidiary cells between knobs (38 cps)).^24–26^ From this data, the authors discussed mechanisms of surface silica deposition (upon epidermal growth after the first asymmetric division of the meristemoid) postulating that silicic acid is secreted through the outer cell wall *via* pores or ectodesmata that might be comparable to those involved in the deposition of cutin on the surface of epidermal cells or cellulose and callose. Relatively recently, fluorescence microscopy has confirmed the role of callose in *Equisetum* silicification.^27^

Since the early identification of silica, it's role in the *Equisetum* life cycle has been explored and the silica-encrusted epidermis was suggested to deter insects, snails,^28^ and other organisms.^29^ However, the cell's mechanical reinforcement was thought to compromise mobility: for example, all movement of the cell walls in older stomata.^25,30^ The considered functions, so far consider *Equisetum* biosilica as a passive mechanical structural element. Addressing the physico-chemical properties of *Equisetum* stomata silica may help better understand what makes this “living fossil” thrive.

The chemistry and biochemistry of silica critically depend on surface charge.^31^ Silica surfaces in aqueous environments carry a negative surface charge, mainly because of the dissociation of terminal silanol groups that occurs at physiologically relevant pH values. This negative charge is counterbalanced by positively charged ions in the electrolyte. Experimentally, in the bulk, this can be studied by potentiometric acid–base titration: an approach that is commonly applied to colloidal dispersions.^31^ Alternatively, characterization of silica ionization may be conducted using microelectrophoresis,^32,33^ streaming potential measurements,^34^ conductometry,^35^ electroacoustic methods^36^ and zeta potential measurements.^37^ However, all these methods rely on approximate models for electrostatic or hydrodynamic processes in the interfacial region. In this respect, being able to characterize charge directly and at the nanoscale, there is interest in microscopy-based approaches such as Kelvin Probe Force^38^ and Atomic Force^39^ techniques. An alternative method to probe silica charge states in relation to structure is to use a silicaphilic dye in conjunction with confocal emission microscopy.^40^ The approach relies on the sensitivity of a fluorescent molecular probe such as PDMPO(2-(4-pyridyl)-5-((4-(2-dimethylaminoethylaminocarbamoyl)methoxy)phenyl)oxazole) to silicon,^41^ silanol and siloxide moieties:^42^ the molecule has been reported to demonstrate pH dependent chromaticity in water, and when in the Stern layer next to silica surfaces.^43^

**Fig. 1:**
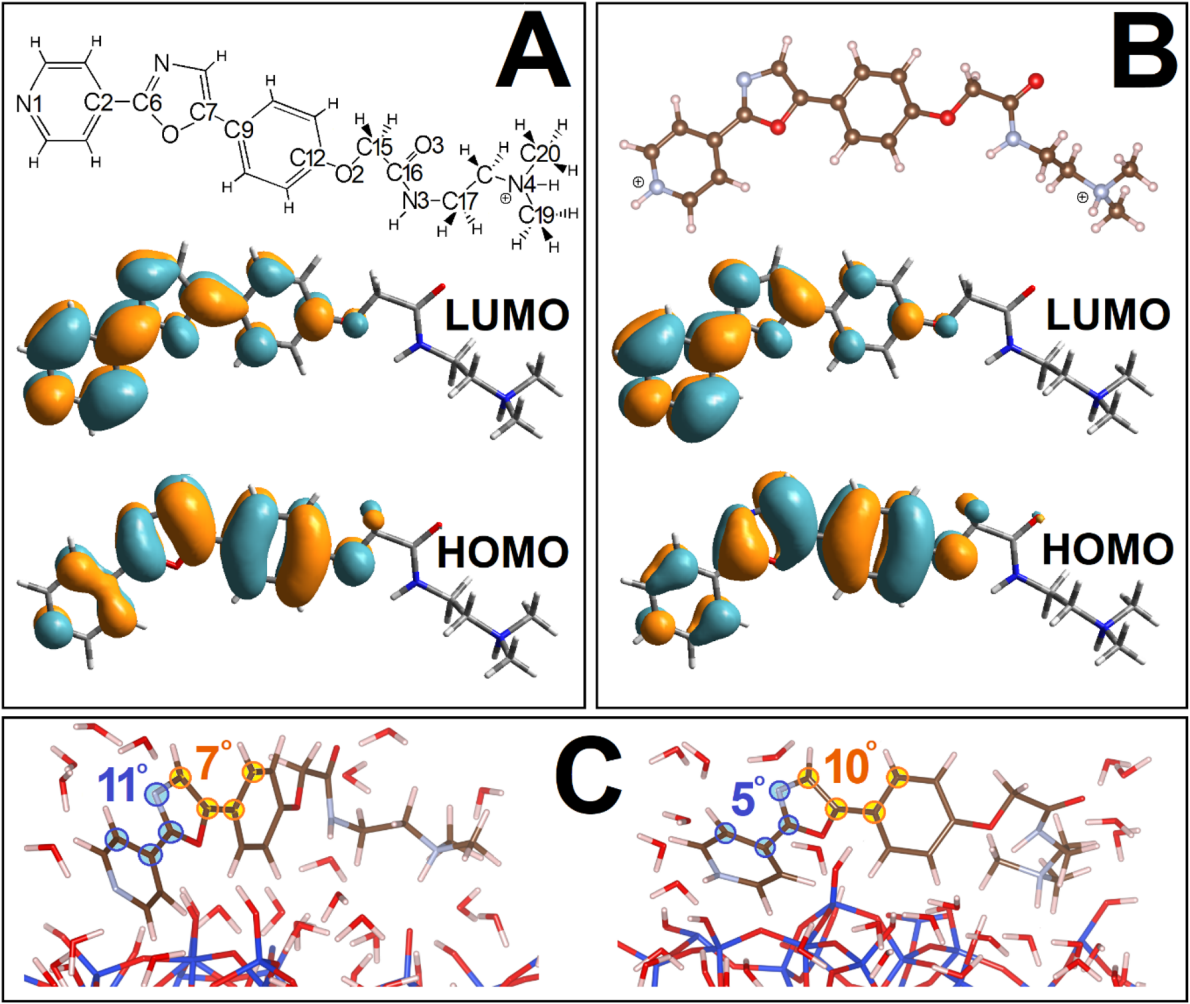
(A) Structural layout as well as HOMO and LUMO computed using TDDFPT for PDMPOH^+^ chromophore in vacuum. (B) Optimised structure, and frontier orbitals computed for PDMPOH_2_^2+^ chromophore in vacuum. (C) Images of exemplary cases of silica associated PDMPOH^+^ and PDMPOH_2_^2+^ while distorted in respect to energy global minimum flat structures, as computed in vacuum.

Since we aim for understanding of the PDMPO chromophore as a universal probe for biosilica interfaces, we need to account for typical pH variances in water, soil and in biologically relevant media. For example, while the average pH of the ocean surface may vary between 8.15 to 8.05,^44^ degradation of organic matter may lower pH down to 7.8 in deep waters,^45^ and biological productivity at the surface may raise pH up to 8.4.^46^ Comparatively, many river and lake waters demonstrate pH that varies in the range 6–9, depending on geological, biological, and climatic factors.^47^ Next, while soil chemistry reports pH to vary from 3.5 to 9, in terrestrial environments, there is a larger diversity of calcicole species (occurring chiefly on chalk and limestone where pH > 7) than calcifuge species.^48^ Finally, the pH in different compartments of an animal body may demonstrate values between 1.5 and 3.5 for gastric acid,^49^ 4.7 in human skin 50 and to vary between 7.34 and 7.45 for blood.^51^ Considering the typical biosilica pH range to vary from 3.5 to 8.4, in this study, we address properties of single PDMPOH^+^ and doubly PDMPOH_2_^2+^ protonated PDMPO,^40,43,52^ as shown in Fig. 1A1 and A2.

According to the introductory studies, using this molecular probe, it is possible to distinguish different ionic states at the surfaces of an ancient silicified plant, *Equisetum arvense* and an exemplar Antarctic diatom *Nitzschia stellata* at resolutions typical for emission confocal scanning microscopy.^40^ In the biological tissues evaluated using the approach, effective zeta potential was correlated with the ratio PDMPOH_2_^2+^/PDMPOH^+^ according to the fluorescence response integrated over wavelength ranges 500–510 and 460–470 nm respectively. Here, distinction of local ionic character is according to our understanding of the electronic properties of the molecular probe at the surface in relation to the local environment. To understand and develop the approach further it is important to review interactions occurring at the molecular level. We need to model the structure and electronic properties of molecules at silica and account for the quantum nature of the interacting moieties and any dynamics occurring, including proton transfer.

In this contribution, using the silicaphilic PDMPO fluorescent probe, we introduce three-dimensional ionic tomography under sub-micron resolution to address chemical properties of *Equisetum* stomata silica that could have aided the competitive survival of the plant. To understand selectivity of the probe, we model PDMPO chromophores at hydrated silica surfaces employing Born–Oppenheimer quantum dynamics simulations and density functional theory (DFT). Accordingly, we characterise a range of molecular scale structures with promising potential to describe interactions with local silica moieties, address proton transfer events, evaluate energetics of different binding regimes, visualize perturbations of surface electronic densities, compute optical transitions and define the optical properties of the dye when bound to silica in the visible and near infrared spectral ranges.

Applying this theory, we review the pH dependence of PDMPO emission when in water and in the presence of silica. Comparing observed and computed emission spectra for PDMPO when next to silica under different levels of protonation, we compare maps of ionic distributions under single-colour and two-colour excitation regimes. Constructing sub-micron tomographic presentations for the low ionic silica sites, we provide an update to the structural model of silica distribution in the specific subsidiary cells of *Equisetum arvense*. Also, comparing the relative distributions of high and low ionicity sites for closed and open stomata we bring to attention a possible role of stomata silica to moderate methane and carbon dioxide transport. Finally, we discuss next perspectives and the promise of confocal microscopy to sample the ionic character of a silica interface at the local level.

## Methods

### (1) Sample preparation

Solutions of PDMPO (LysoSensor™ yellow/blue DND-160) in dimethyl sulfoxide (1 mM) were obtained from Life Technologies. We collected plants of *Equisetum arvense* from the banks of the Leen River and in the fields of Clifton, Nottinghamshire. For confocal microscopy, we sliced stems with epidermis from different positions on internodes and placed samples in PDMPO solution at 1 μM for 24 h, and longer.

### (2) Emission spectroscopy and microscopy

Details of emission spectral measurements using 96-well plates (Nunc optical bottom plates (164588)) using a commercial microplate reader (M200 pro; Tecan) were reported previously.^43^ We conduct fluorescence imaging using a Nikon AX confocal microscope (Nikon Europe) with a PLAN APO λD 20×/0.8NA objective. We set the XY galvano unit of the microscope with a 4 μs per pixel dwell time to scan images of 1024 × 1024 pixels (along *x* and *y* directions) with spatial resolution by 0.168 microns per pixel, and intensity under 14 bit digital conversion. Data from two channels were collected with 405 nm (15%) excitation and emission recorded using a GaAsP PMT with a gain of 30 using a DUX-VB detector over 460–470 nm and 500–510 nm with a quad band dichroic as the main beam splitter and a linear variable filter for emission selection. The third channel used 561 nm (3%) excitation laser with emission collected over 600–750 nm with a 20/80 beam splitter used to avoid the 640 nm region block of the quad band dichroic. A pinhole of 18.3 microns was used corresponding to 0.8 airy units, with a *z* step of 1 micron.

For each sample, we conduct two experiments. In the first experiment, we adopt excitation at 405 nm to collect images in two spectral ranges: between 460 and 470 nm, and between 500 and 510 nm. As a first approach, we ascribe the images to PDMPOH^+^ and PDMPOH_2_^2+^, respectively. Using two images detected under the same excitation, we follow the ratiometric processing, as described previously (save, without resolution reduction but using Fourier transform filtering) to express ionicity maps.^40^ Since we use the same excitation wavelength for both sets of emission data, we address the collected results as of a single-color experiment.

In the second experiment, we excite at 532 nm to collect images in the spectral range of 600–750 nm. This experiment takes account of suggestion from theory, that under excitation at 405 while detecting in the spectral range of 500–510 nm (which we ascribe to be specific to PDMPOH_2_^2+^), there could be a slight contaminating contribution from PDMPOH^+^. To cross-check the quality of the ionicity map we generate using the results of the single-color experiment, here, upon ratiometric processing we use the image detected within 460–470 nm under 405 nm excitation, and an image detected with emission at 600–750 nm under 532 nm excitation as signatures specific to PDMPOH^+^ and PDMPOH_2_^2+^, respectively. Since the excitation wavelength is different, we address the collected results as of a two-color experiment.

### (3) Theoretical studies

In contrast to structural extracts of the chromophores alone under stationary DFT optimization in vacuum,^43^ we may expect a significant diversity of structural forms when next to silica surfaces having different charge. To allow for this, our computational approach is as follows: first we sample variations in PDMPO dynamics using a semi-empirical (tight binding) approach in (a) vacuum, (b) in explicit water and, subsequently, (c) when next to a monolayer of silica, which is prepared in a neutral form (no charge) or with ionic, charged defects. Second, considering that experiments are conducted at silica in the solid state; to refine electronic and structural properties and for selected structural realizations, we consider a two-layer silica structure built by adding a second silica layer below the original monolayer, according to the geometry of α-cristobalite,^53,54^ and we conduct quantum *ab initio* Born–Oppenheimer molecular dynamics (BOMD) thermalization dynamics followed by a short trajectory to sample conformational averages about the received mineral structure. This approach allows us to address the complexity of the configuration space for large chromophores next to a hydrated silica surface, where local ionic sites affect binding. In other words, after sampling fast structural variances at a silica monolayer using tight binding dynamics, we improve relevance with the experiment by carrying out BOMD on selected structures at silica updated with second layer. Third, for the selected structural cases of the chromophores at the double-layer silica, we conduct structural optimization, electronic properties modelling and computation of optical transitions using DFT at the same level as we used for BOMD. In this contribution, we perform all theoretical studies using the CP2K software package.^55^

To model the structural and electronic characteristics of PDMPO next to silica, we use a Q^3^ surface (where each silicon atom is attached to three silicon atoms *via* oxygen bridges and one hydroxyl group) which may be considered as representative of amorphous silica and porous glasses.^56^ As reported previously, this may be obtained by a cleavage along the (101̄) plane of α-cristobalite^53,54^ followed by hydration of dissociated bonds.^57^

First, we adopt a tight-binding approach^58^ to model structural variances of the chromophores in vacuum and in water (91 explicit molecules) initialized in an orthorhombic supercell (23.9 × 31.6 × 22.0 Å) under a periodic boundary condition and using a Langevin thermostat. After thermalization, we sample 5 ps long trajectories with a step of 0.5 fs.

Next, we conduct tight binding molecular dynamics simulations for PDMPO chromophores next to a silica monolayer. Specifically, we select several structural cases of the two chromophores to place above a single silica layer (48 Si atoms according to (101̄) plane geometry) in the presence of 64 water molecules to fit an orthorhombic supercell (16.5 × 29.6 × 40.0 Å) under periodic boundary conditions. We condition the prepared surface (with area of 4.889 nm^2^) either to be neutral (S0), where all terminals are silanol groups, or to include a single siloxide moiety (S1), or to present two siloxide groups (S2). Considering the surface area, introduction of one or two anionic sites provides 0.205 and 0.41 siloxide per nm^2^, respectively. According to previous experimental studies, such a presence of siloxide sites is observed for the pH range from 4 to 6.^52,56,59^ Using a Langevin thermostat we thermalize the prepared systems to sample 5 ps trajectories with a step of 0.5 fs.

Second, based on the results of the tight binding molecular dynamics simulations for PDMPO chromophores next to the silica monolayers, we selected three representative cases of PDMPOH^+^ at neutral silica, at silica with one anionic site next to the dimethylamine moiety, and at silica with two anionic sites proximal to both, the dimethylamine moiety and the pyridine group. Additionally, we selected nine structural cases of PDMPOH_2_^2+^ when next to silica: three cases when at a neutral surface, three structures at silica with one anionic site next to the dimethylamine moiety, and three systems at silica with two anionic sites proximal to both, the dimethylamine moiety and the pyridine group. Accounting for the nature of silica in experiments, to improve the modelling of the electronic structural properties of the selected structural model cases, we update the silica substrate by adding a second (lower) layer according to α-cristobalite geometry.^53,54^ To be able to compute the structures, we reduce the total number of atoms to *c.a.* 500 removing extraneous water molecules, keeping only those that are proximal to silica and PDMPO. It is important to note here that an excess of water surrounding PDMPO would make computing optical properties of the system unfeasible due to a continuum of electronic transitions to include contributions of aqueous states. Next, to relax the two-layered structures, we conduct BOMD simulations. We use the Perdew–Burke–Ernzerhof exchange-correlation functional,^60,61^ Grimme D3 contribution,^62^ and double-ζ polarization basis^63^ for Geodecker–Teter–Hutter pseudopotentials.^64^ The cut-off and the relative cut-off of the grid level are set to 280 and 60 Rydberg, respectively. According to this setting, we conduct thermalization under the velocity rescaling regime,^65^ and consequently perform 50 fs NVT sampling under a NOSE thermostat.^66^

Third, considering the results of BOMD, we select 9 representative structural cases to optimize structures using density functional theory (DFT) under CP2K GEO_OPT criteria of 0.001, and express optical electronic transitions employing time-dependent density functional perturbation theory (TDDFPT).^67^ To discuss computed properties and relevance to physical experiments, further, in the main text, we detail properties of six structural realizations: 3 cases of PDMPOH^+^ and 3 cases of PDMPOH_2_^2+^ next to S0, S1 and S3 surfaces respectively. The ESI† contains properties of the other systems.

In the following section we examine the results of molecular dynamics simulation and density functional theory on structural realizations, electronic and optical electronic properties of PDMPO chromophore when next to various silica surfaces. Assisted with theory insight, we review experimental dependences of PDMPO emission on pH when in water and next to silica. To explain the observed spectral dependencies, we detail molecular mechanisms of PDMPO sensitivity to silica surface ionicity. Accordingly, we adopt informative spectral regions to conduct sub-micron ionic tomography of *Equisetum arvense* stomata and discuss the role of bioinorganic silica in stomata functionality.

## Results

### (1) Structure and electronics in vacuum

The sensing capacity of the PDMPO chromophore at silica is due to modulations of its structural–electronic properties, in response to variances at the mineral interface. To model mechanisms of such interactions, first, using DFT, we optimize structure and compute electronic properties of the dye in vacuum. Upper images in Fig. 1A and B present a structural layout for PDMPOH^+^ and a visual presentation for the optimized structure of PDMPOH_2_^2+^, respectively. Theory anticipates both chromophores to demonstrate ideally flat geometry for the aromatic component and the ether connected amide unit.

Using TDDFPT we address the optical electronic properties of the chromophores. In Fig. 1 we compare HOMO and LUMO for the two molecules. While the experiment using titration^43^ reports the lowest UV resonances for PDMPOH^+^ and PDMPOH_2_^2+^ to be at about 331 and 382 nm, respectively, theory anticipates the corresponding optical transitions at 417 and 460 nm, respectively. Exploring the frontier orbitals, one may clearly see, theory suggests that the aromatic moiety of the chromophore (from atom N1 to atom C12) governs the red edge optical electronic properties. While the frontier orbitals may not be most helpful to visualize electronic redistributions upon excitation, charge transfer contributes into the first transitions. Accounting for the structure of the molecules in the Franck–Condon region, our theoretical studies suggest that asymmetry of the optical spectra as we reported in ref. 43 may be ascribed to vibronic contributions. The ESI† details charge transfer numerical evaluations, as well as comparisons of experimental and theoretical spectra computed accounting for vibronic progressions in the Franck–Condon region.

Next, using the tight binding approach,^58^ we address dynamics of the chromophores in vacuum, in water and at a hydrated silica monolayer. The geometry of the aromatic moiety is determined by the torsion angles about the C2–C6, C7–C9 and O2–C12 bonds (Fig. 1A). Both, in vacuum and in water, the angles fluctuate (in the range ±50°) about 0° to preserve more or less the flat geometry of the aromatic group. When next to silica, the angular distribution broadens and asymmetry is present in respect to the means of structures computed in vacuum, see Fig. A3, A4, B4 and D3 in the ESI.† Interactions and association with the surface stimulate both, departure of the aromatic moiety from the flat geometry and asymmetry of the molecular frames. Representative structures are presented in Fig. 1C. Description and discussion of the rotation dynamics are provided in the ESI.†

### (2) PDMPO protonation: simulations of hydrogen bond dynamics when at silica

**Fig. 2:**
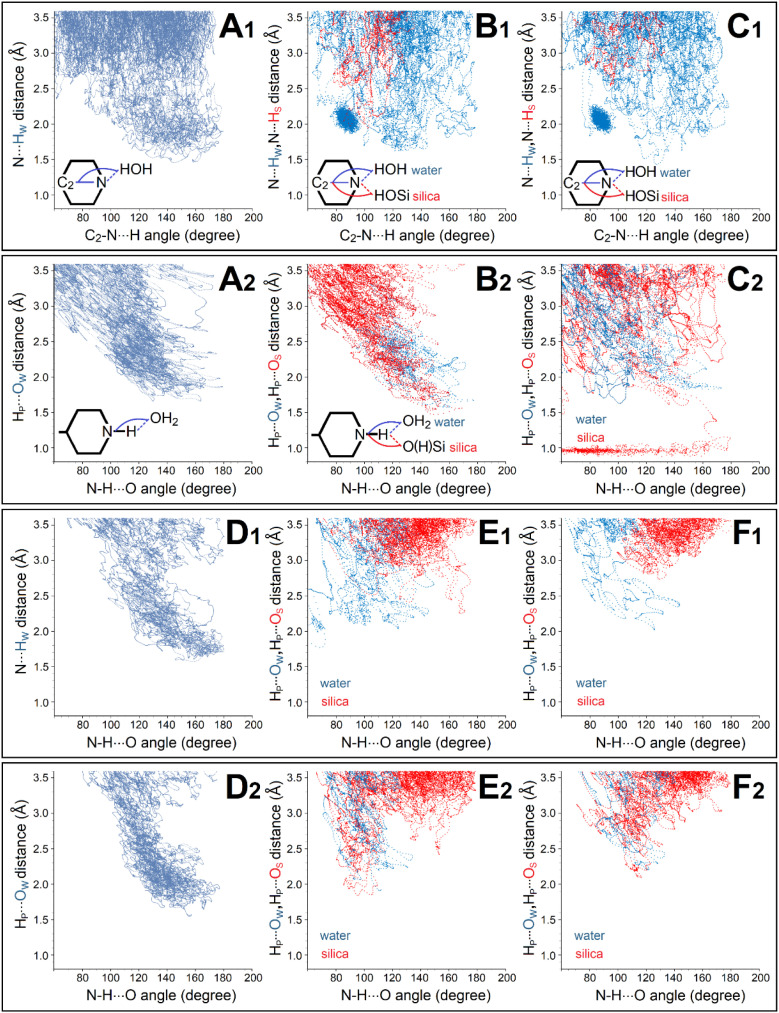
Hydrogen bond dynamics computed by tight binding molecular simulations for PDMPO chromophores in water and next to a silica monolayer: ESI† provide an extended data set. Characteristics of hydrogen bond dynamics involve the pyridine moiety of PDMPOH^+^ chromophore when in water (A1), when next to silica with a siloxide moiety proximal to dimethylamine (B1), and next to silica with two siloxide groups proximal to both sides of PDMPOH^+^ (C1). Characteristics of hydrogen bond dynamics involve the dimethylamine moiety of PDMPOH^+^ chromophore in water (D1), when next to silica with siloxide moiety proximal to dimethylamine (E1), and next to silica with two siloxide groups proximal to both sides of PDMPOH^+^ (F1). Panels A2–F2: characteristics of hydrogen bond dynamics involve the corresponding moieties of the PDMPOH_2_^2+^ chromophore. Blue and red dotted lines present geometric properties specific to hydrogen bond dynamics upon interaction with water and silica, respectively; subscripted letters W and S in ordinate axes labels indicate atoms of water and silica, respectively.

Polar interactions at the interface dominate in defining the structural forms of PDMPO at silica. Allowing for the complexity of the configurational space when at a silica surface we may search for energy minima of relatively stable arrangements while exploring intermolecular geometric patterns specific to hydrogen bonding of different types. In Fig. 2 we explore results of tight binding molecular dynamics to evaluate hydrogen bond dynamics for PDMPOH^+^ and PDMPOH_2_^2+^ chromophores in water and next to S0, S1 and S2 silica monolayers, while Fig. S6 in the ESI† provides an extended set of data to account for other cases. The chosen configurations have been proposed to play a decisive role in resolving bio-silica local charge using microscopic sampling of PDMPO emission^40^ in the range of pH typical for living matter. To model binding cases, first, we characterise hydrogen bond dynamics that involve the pyridine of the PDMPOH^+^ chromophore: we sample the distance between the pyridine N atom and proximal H atoms of water (blue dotted line) or of silica (red dotted line) *versus* angle between the nitrogen atom and HO moiety of water or of silica: see panels in Fig. 2A1–C1. Analogously, for the PDMPOH_2_^2+^ chromophore, where the pyridine is protonated, we extract the distance between the pyridine H atom and proximal oxygen atoms of water (blue dotted line) or of silica (red dotted line) *versus* the angle between the hydrogen atom and OH moiety of water or the O–Si structural element of silica: see panels in Fig. 2A2–C2. In order to describe hydrogen bonding involving the dimethylamine group of both, PDMPOH^+^ and PDMPOH_2_^2+^ chromophores, we sample distances between the hydrogen atom of the group and proximal oxygen atoms of water (blue dotted line) or of silica (red dotted line) *versus* the angle between the hydrogen atom and OH moiety of water or OSi structural element of silica: see panels in Fig. 2D1–F1 and D2–F2.

The data simulated for the PDMPO chromophores in water indicate effective hydration of its pyridine moiety with first neighbour hydrogen bond lengths ranging from about 1.5 to 2.7 angstrom: see Fig. 2A1 and A2. Simulations for the PDMPOH^+^ chromophore next to silica specify that for this distance range (up to 2.7 angstrom), water dominates in hydrogen bonding. Red trajectories in Fig. 2B1 and C1 show that distances between pyridine nitrogen and silica hydrogens are longer, on average, than such between the pyridine nitrogen and water hydrogens (blue trajectories). At the same time, for the PDMPOH^+^ chromophore next to silica, theory suggests a narrow clustering of water next to the nitrogen of pyridine: see the narrow blue distributions that peak at about 2 angstroms in Fig. 2B1 and C1. The preferred orientation corresponds to waters attracted to the N1 atom under orthogonal orientation in respect to the aromatic plane of the molecule as shown in Fig. 1A1. Additionally, for PDMPOH^+^ the partial Mulliken charge on the N1 atom is −0.217. Upon protonation, the chemistry of pyridine is substantially different: the Mulliken charge on the nitrogen is 0.296, and its proton allows hydrogen bonding with oxygen either of water or silica. In the case of the deprotonated pyridine of PDMPOH^+^, its hydrogen bonding is according to relatively fast rearrangements of a proximal H^+^ network. In the case of the protonated pyridine of PDMPOH_2_^2+^, its hydrogen bonding is in dependence on slower intermolecular motions to provide a contact with a suitable oxygen. In this respect, it is interesting that Fig. 2B2 and C2 present computed results that associations with silica (red trajectories) dominate over the interactions with water (blue trajectories). Also, due to the different electronic nature of the protonated pyridine, there is no longer a structurally narrow water clustering next to it, as theory suggests when it is deprotonated: Fig. 2B1 and C1.

Another interesting effect of pyridine protonation concerns orientation constraining for the first neighbour interactions. Fig. 2A1–C1 demonstrate uniform distributions for PDMPOH^+^, while Fig. 2A2–C2 present off-diagonal correlation tendencies in the distance-angular maps for PDMPOH_2_^2+^. Such tendencies are somewhat smeared when there is a siloxide group next to the protonated pyridine: Fig. 1C2. Therefore, theory anticipates a narrower configuration space for intermolecular relations of the pyridine moiety of PDMPOH_2_^2+^ either in water or next to silica. We may ascribe the angle–distance intermolecular correlation as modelled for the PDMPOH_2_^2+^ chromophore to introduction of an electric dipole to the pyridine moiety upon its protonation: the charge of the N1 atom becomes positive, while the negatively charged C2 is weakly sensitive to protonation. It is curious that when a siloxide group is next to the protonated pyridine, according to the adopted theory, the system may demonstrate proton transfer: note the red dotted line shows a decrease of the distance between the proton of pyridine and an oxygen of silica. As we discuss later, relative flexibility of H^+^ between pyridine and silica may determine the sensorial capacity of this silicaphilic chromophore to report on local ionicity changes to its emission properties.

In respect to hydrogen bond dynamics for the dimethylamine group, both, PDMPOH^+^ and PDMPOH_2_^2+^ chromophores demonstrate analogous angle–distance distributions when in water: see Fig. 2D1 and D2. The character of the structural distributions is sensitive either to the dimethylamine group of PDMPOH^+^ or PDMPOH_2_^2+^ when they experience a siloxide moiety nearby. In the case of the PDMPOH^+^ chromophore (Fig. 2E1 and F1), water dominates in first neighbour interactions. However, silica prevails in the second neighbour interactions. In the case of the PDMPOH_2_^2+^ chromophore (Fig. 2E2 and F2), interactions with silica become equally competitive or even dominant. Theory suggests that the bulky methyl group may play a significant role in aqueous screening of silica sites. Once again, here: Fig. S6 in the ESI† provides an extended data set addressing hydrogen bond dynamics of PDMPO chromophores at silica of different ionicity.

### (3) PDMPO silicaphilic nature: energetics and electronics of binding

**Fig. 3:**
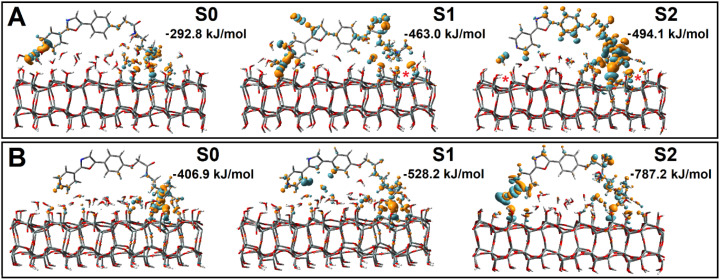
Perturbations of electronic density upon binding of PDMPOH^+^ (top) and PDMPOH_2_^2+^ (bottom) chromophores to neutral silica (S0), silica with one siloxide group next to dimethylamine (S1), and two anionic silica sites next to pyridine and dimethylamine (S2). Red stars in the top panel indicate approximately the locations of the siloxide groups. Binding energies are for structures optimized after removal of water molecules, see the main text for details.

Sampling various PDMPO structural realizations at silica, we would like to evaluate, approximately, how strong is the energy gain for PDMPO on association with silica under different protonation conditions and what happens to the electronic properties of PDMPO and silica when they interact. Such information would characterize (overall) the mechanisms that make PDMPO silicaphilic. Information presented in Fig. 2 is pivotal in selection of structural cases for further detailed studies of PDMPO interactions with silica. To model properties with relevance to experiments at solid silicon oxide,^40,43^ for the extracted structural cases (overall, sixteen promising arrangements) we add a second silica layer while matching α-cristobalite geometry^53,54^ and relax structural stress (induced upon the second layer addition) conducting BOMD thermalizations, as we describe in the Methods section. DFT theory was used to optimize structures and explore how binding affects the electronic properties of the surface and of the chromophores. Specifically, in Fig. 3, we present images of electronic density changes for selected structural cases next to S0, S1 and S2 surfaces, while Fig. S7 in the ESI† provides images and binding energies for the wider scope of explored structural cases.

For both chromophores at the neutral surface, where the dimethylamine group is proximal to the surface, theory indicates moderate perturbative increase of electron density for oxygens of the silanol groups and reciprocal decreases of electron density on the nearest hydrogens of the dimethylamine group: see corresponding cyan/orange colored 3D components in Fig. 3. For the neutral surface, the pyridine moiety does not show strong interactions with silica, but with water that is close by. When next to silica with one siloxide near the dimethylamine moiety (S1), the extent of electronic perturbation on silica components (next to the anionic defect), proximal water and skeletal moieties of both chromophores is larger. For the PDMPOH^+^ chromophore, theory anticipates strong perturbation along the hydrogen bond between a silanol and the nitrogen of the pyridine group. In the case where the chromophores are next to silica with two anionic sites, the extent and amplitude of the electronic perturbation is the largest, which is in general agreement with the modelling of interacting charges.

Having visual comparisons, next, we estimate energetics of binding. To manage this, we conduct an additional series of theoretical studies. Specifically, we review each structural case of interest, remove water molecules which do not participate in bridging between the PDMPO chromophore and silica, and conduct structural optimizations for such systems. Consequently, for each optimized case, we compute energy for silica (after we remove PDMPO atoms) and PDMPO (after removing silica and associating water with it, to maintain its bridging contribution). In Fig. 3 we list the corresponding binding energies. Here, it is important to stress, that to evaluate interaction energy between chromophore and silica, we conduct binding studies at essentially dry interfaces: for each structural case, we remove interfering water and reoptimize the structure. Therefore, the binding energies (we indicate numerical values for each case as presented in Fig. 3), for the initial hydrated cases, are only to compare tendencies for binding between the cases. We may see that neutral silica is somewhat attractive to both protonated charged forms of PDMPO. Of course, binding energy increases with silica ionicity. Fig. S7 in the ESI† provides images and binding energies for the wider scope of explored structural cases. Overall, theory suggests a significant variance of prominent energetics for PDMPO binding to silica surfaces in dependence on the nature of the silica surface and local acidity.

### (4) Computed optical electronic properties

**Fig. 4:**
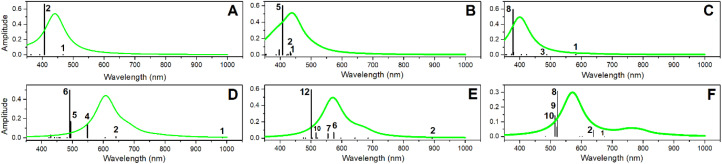
Optical absorptions resonances (black lines) computed for the selected structural cases of PDMPOH^+^ and PDMPOH_2_^2+^ chromophores. (A) PDMPOH^+^ next to neutral silica (S0); (B) PDMPOH^+^ with tail next to silica anionic site (S1); (C) PDMPOH^+^ with tail and head next to two anionic sites (S2). (D–F) Optical properties computed for analogous arrangements of PDMPOH_2_^2+^ next to silica. Emission spectra (green lines) are computed considering the most intense transition, which may be excited using 405 nm radiation, as employed in confocal microscopy experiment.^40^

We now consider the optical properties of PDMPO chromophores when next to silica. In Fig. 4 (see black vertical lines) we present frequencies and amplitudes of optical absorption resonances for the considered systems. Fig. S8 in the ESI† presents an extended set of computed optical absorption spectra. One may clearly see that theory, at the adopted level, estimates optical absorptions of PDMPOH^+^ and PDMPOH_2_^2+^ chromophores to peak at about 400 and 500 nm, respectively.

The spectral difference of the optical absorption maxima is comparable to that reported for the two chromophores according to experimental results in buffers.^43^ In respect to the computed wavelengths, it is necessary to note that optical absorption of PDMPO when not bound to silica is significantly shifted into the UV spectral range: again, this is according to experimental spectral studies.^43^

Additionally, in Fig. 4, using green lines we present emission spectra computed for the considered systems. According to theory, the maxima of the emission spectra computed for PDMPOH^+^ and PDMPOH_2_^2+^ chromophores have peaks at 450 and 500 nm, respectively. This is in reasonable agreement with experimental data.^43^ Here it is important to note that we were able to reproduce experimental spectra optimizing structure and computing optical properties for the excited states which are involved in optical transitions of maximal oscillator strength. For example, for the PDMPOH_2_^2+^ at silica S0, emission arises from optical transitions specific to the excited state 6, while for PDMPOH_2_^2+^ at silica S2, we sum emissive dispersions for the system in the excited states 10 and 9, to demonstrate dominant oscillation strengths, when the system is optimized in the excited states 8, 9 and 10. To reproduce the experimental spectra, theory suggests that the visible spectral range emission for the modelled systems does not come from the lowest excited state. For a protonated chromophore at silica, TDDFPT computes relatively weak oscillator strengths for the lowest energy transition and a large energy gap to relax to the LUMO: the two mechanisms are distinguished to explain a possible compromise^68^ of Kasha's rule.^69^

Here, it is interesting to note that the wavelength of the maxima of the computed optical absorption and emission for PDMPOH^+^ (and for PDMPOH_2_^2+^) do not show a strong tendency in dependence on the ionicity of silica surfaces. For example, for the case of PDMPOH^+^, one may confirm this comparing green spectra maxima in Fig. 4A–C. Analogously for the case of PDMPOH_2_^2+^, one may observe this comparing green spectra maxima in Fig. 4D–F. At the same time, theory indicates that the nature of states contributing into the emission maxima of PDMPOH^+^ and of PDMPOH_2_^2+^ is different. Theory suggests that for both, PDMPOH^+^ and PDMPOH_2_^2+^ the higher numbered energy transitions determine optical absorption and emission maxima. Taken together, the lower energy optical absorption transitions tend to shift to the near infrared spectral region, when the chromophore is next to silica with an anionic site. This is more obvious for PDMPO(H^+^): compare frequencies of the lower absorption transitions in Fig. 4A–C.

**Fig. 5:**
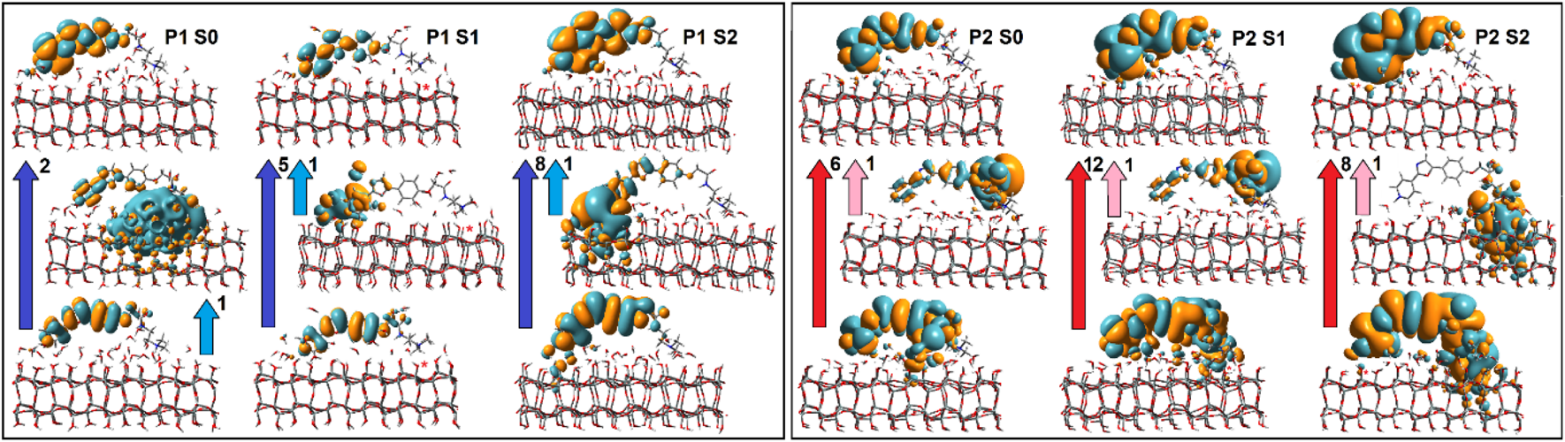
Molecular orbitals of the first (pale blue and pale pink arrows) and the most intense transition (dark blue and dark pink) in the visible spectral range (see Fig. 4) as computed for selected structural cases of PDMPOH^+^ (left) and PDMPOH_2_^2+^ (right) chromophores next to different silica surfaces, as indicated.

To understand the underlying physics better, in Fig. 5, for the considered cases, we present molecular orbital components specific to (a) the first (lowest) energy transition (pale blue and pale pink arrows) and (b) the transition with the largest oscillator strength (dark blue and dark pink/red arrows). The computed properties indicate the different nature of the electronic states for PDMPOH^+^ (blue arrows) and PDMPOH_2_^2+^ (pink arrows) chromophores when next to silica.

Specifically, in the case of PDMPOH^+^ next to S0 and S1 surfaces, the electronic departure states of the first transition (where HOMO contributes significantly) include no contributions from silica and very weak admixing with silanol *via* interactions with the pyridine side of PDMPOH^+^, respectively. When the PDMPOH^+^ chromophore is next to a S2 surface, theory suggests moderate admixing of pyridine electronics with that of a proximal silanol *via* nearby water molecules. Concerning the nature of the lowest excited states, theory suggests a weak admixing of PDMPOH^+^ and silica electronic components. This, however, is prominent for the first transition when the chromophore is next to the uncharged S0 silica surface. In respect to the nature of the higher excited states for transitions of maximal intensity, in all cases, theory indicates no admixing of PDMPOH^+^ and silica electronic components.

In contrast, when the PDMPOH_2_^2+^ chromophore is next to silica, regardless of the ionic charge carried, the electronic departure state of the first transition involves a mixing of PDMPOH_2_^2+^ and silica electronic components. Here, in the electronic departure states, both, pyridine and dimethylamine moieties participate in interactions with silica, though, the latter plays a dominant role. Concerning the nature of the excited states, in all cases, theory indicates rather effective admixing of PDMPOH_2_^2+^ and silica electronic components *via* the pyridine moiety.

To conclude, in the case of the chromophore in the PDMPOH^+^ state, theory indicates pyridine-to-silica electronic redistributions determine optical properties in the visible spectral range. In the case of the chromophore in the PDMPOH_2_^2+^ state, typically, the lowest energy transitions (in the near infrared) describe charge redistributions from the dimethylamine to the pyridine side of the chromophore alone. At the same time, charge transfer contributions from silica to PDMPO dominate the most intense transitions in the visible spectral range.

Having computed optical electronic properties of a PDMPO chromophore at silica under the selected representative arrangements, next, we need to describe the molecular mechanisms of PDMPO selectivity as a probe of local silica ionicity. To approach this, we must account the structural properties and interactions of PDMPO and silica in dependence on ionicity and protonation. First, according to theory, in contrast to the rather flat geometry of PDMPO chromophores in vacuum and in water, when next to silica interfaces, PDMPO is expected to show a variety of distorted conformers with intermolecular arrangements under constraining correlations in dependence on the protonation state of the chromophore. In our studies, as an introductory approach, we adopt a relatively flat silica surface based on the geometry of the (101̄) plane of cristobalite. Such a surface, however, demonstrates a pattern of silanol and siloxide moieties sufficient to create an interfacial force field, which moderates the anticipated departure of PDMPO conformation from its flat geometry in vacuum. In experiment, silica surfaces are far from ideally flat. Accordingly, both, flexibility of the dimethylamine anchor in respect to the aromatic group and the distinct electronic properties of the two sides of the chromophore are critically important for chromophore–silica binding.

Second, adopting molecular dynamics under both, tight binding and the Born Oppenheimer regime, as well as stationary DFT, in a number of structural cases, we model proton transfer from the pyridine moiety of the chromophore as PDMPOH_2_^2+^ when next to a siloxide group. Our studies suggest that such proton transfer dynamics is less likely when the siloxide is involved in a hydrogen bonded network to include water molecules and nearby silanols. This corresponds with the discussion in a previous theoretical report,^70^ where the authors address involvement of water to define p*K*_a_ of a local site: that the type of a silanol (ether Q2 convex or concave, as well as either Q3 isolated or vicinal) does not play a role in its acidity but a higher solvent accessibility lowers the p*K*_a_ of such a silanol. In this contribution, we may consider three factors to affect proton redistribution: the relative involvement of water, the extent of a local hydrogen bonded cluster around a silanol/siloxide moiety, and how optimal such a cluster is both structurally and energetically. The more extended the partial sharing of protons is (to cause a larger electronic delocalisation), and the more optimal such a cluster is, the stronger smearing of local charges and their fluctuations should be to attenuate a proton transfer redistribution. Accounting this we may ascribe low and high p*K*_a_ silanol species^71,72^ to solitary well-hydrated silanols and to silanol/siloxide vicinal clusters with participation of water, respectively. Variance of optimal structuring and degree of proton sharing and electronic delocalization in such clusters, while sensitive to a type of surface, provide the range of pH (from 4 to 7.5, or most frequently around 6–7) to determine the polarity of a silica surface under physiologically relevant conditions.^73–75^ Since the distinguished role of silica inclusions in various organisms,^1–6^ the aqueous moderation of the diverse and yet unreactive polarity of silica surfaces may be of a primary importance for living matter since early in evolution. To describe molecular mechanisms of PDMPO selectivity to silica surface local ionicity we must now relate simulated structural and optical properties of PDMPO–silica associations with the physical chemistry of such composites in dependence on pH as measured in experiment.

### (5) Explanation of emission pH dependence: PDMPO sensitivity mechanisms

As we are interested in how silica “works” in living matter, as well as how it may inspire engineering,^7–14^ in this contribution, we focus on increasing understanding as to what makes PDMPO emission sensitive to silica,^43^ and what exactly PDMPO reports on silica surface electronics and if this is under microscopic resolution,^40^ in particular. To answer the questions, next, we model the emission of PDMPO in water and in the presence of silica nanoparticles in dependence on pH^43^ and discuss the nature of the emitting states using instructions from our TDDFPT studies.

Specifically, following common equations for a two-step deprotonation process, we specify contributions of emission spectral signatures for PDMPO in dependence on pH as:
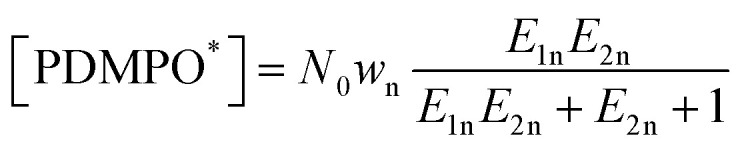

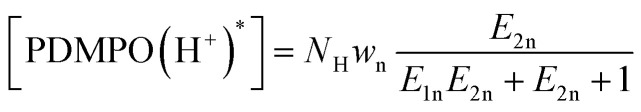

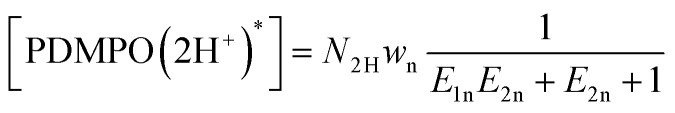
here, first of all, because PDMPO 
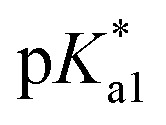
 in the electronic excited state is different from the p*K*_a1_ of the chromophore in the ground state, then, depending on pH, excited PDMPOH^+^* may receive back the second proton from water to recover its double protonated form 
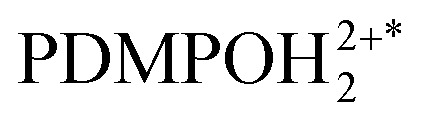
 while in the excited state. Previously, persistence of the emission band at 540 nm for pH > p*K*_a1_ = 4.2 was ascribed to this process.^43^ Since the finite lifetime in the electronic excited state and the time necessary for ambient water to rearrange to transfer the proton, not all PDMPOH^+^* may undergo this process. To account this, we introduce the factor *w*_n_: *w*_n_ = *w* and *w*_n_ = 1 − *w* describe fractions of PDMPOH^+^* chromophores, which do and do not experience proton transfer in the electronic excited state, respectively, when 
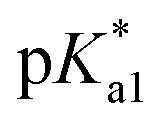
 < pH < 
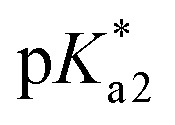
.

For PDMPO forms which do not experience proton transfer in the electronic excited state, *E*_1n_ = exp[p*K*_a1_ − pH] and *E*_2n_ = exp[p*K*_a2_ − pH], *N*_0_ = *A*_0_, *N*_H_ = *A*_H_, *N*_2H_ = *A*_2H_. Here, p*K*_a1_ and p*K*_a2_ are the first and second acid dissociation constants for the chromophore in the electronic ground state, respectively. *A*_0_ and *A*_2H_ present normalized (on sum intensity) emission spectra at pH = 14 and 2.2, respectively; while *A*_H_ presents a normalized (on sum intensity) difference spectrum: spectrum at pH = 7 minus spectrum at pH = 2.2 scaled to remove contribution of the band at 540 nm. For PDMPO molecules, which do experience proton transfer in the electronic excited state, 
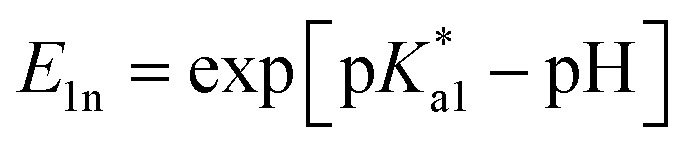
 and 
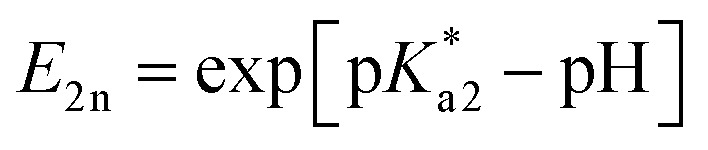
, *N*_0_ = *B*_0_, *N*_H_ = *B*_H_, *N*_2H_ = *B*_2H_. Here, 
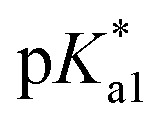
 and 
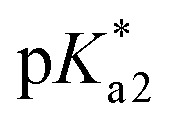
 are the first and second acid dissociation constants for the chromophore forms in the electronic excited state, respectively. For PDMPO in water experiments, *B*_0_, *B*_H_ and *B*_2H_ are the same spectra as *A*_0_, *A*_H_ and *A*_2H_, though, their dependences on pH are according to 
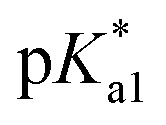
 and 
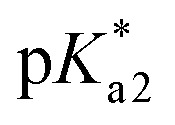
. The ESI† provides details on model derivations and fitting.

**Fig. 6:**
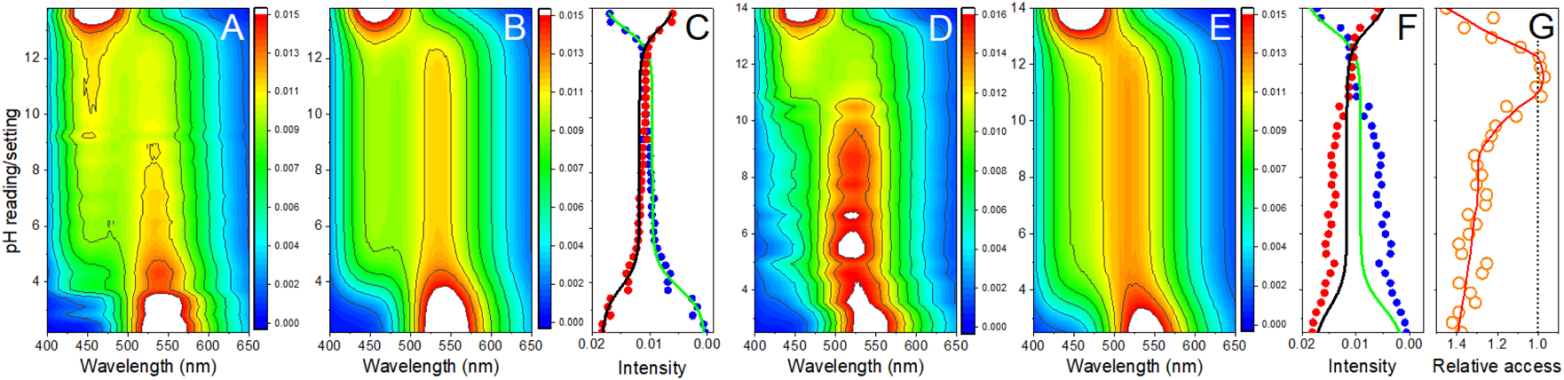
Modelling of PDMPO emission as a function of pH. (A) Normalised emission spectra of PDMPO in water in dependence on pH: each spectrum is normalized on its total intensity. (B) Modelling of PDMPO emission spectrum in water. (C) Comparison of pH dependent emission at 442 and 538 nm (blue and red circle lines) with the correspondent dependences by the model (green and black lines). (D) normalised emission spectra of PDMPO in presence of silica nanoparticles. (E) Application of the “aqueous” model (attuned to describe emission of PDMPO in water) to reproduce data in panel D. (F) Comparison of emission at 442 and 538 nm (blue and red circle lines) of PDMPO in presence of silica with the correspondent dependences using the “aqueous” model (green and black lines). (G) Relative accessibility to proton transfer (in electronic excited state) in dependence on pH (orange circles) computed using experimental and modelled dependences as shown in panel F: red line presents interpolated values.

According to the described model, for p*K*_a1_ = 4.2, p*K*_a2_ = 13.8, 
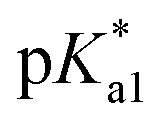
 = 13.8, 
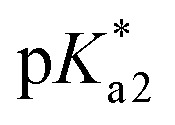
 = 14 and *w* = 0.45, in Fig. 6B we show that the pH dependence of PDMPO emission on pH can reproduce an experimental data set, as we show in Fig. 6A. In Fig. 6C we compare selected vertical slices from panel A, which present pH dependent emissions at 442 and 538 nm (blue and red circle lines) with the correspondent dependences by the model (green and black lines) taken from panel B. For details of the fitting see ESI, Fig. S9–S18.†

Having reasonable agreement between experiment and theory of PDMPO emission in water, we may address the experimental properties of the chromophore in the presence of silica nanoparticles: see Fig. 6D. The experimental data demonstrate a blue shift of the emission when pH changes from 3 to 3.6. In this range of pH, since the chromophore p*K*_a1_ = 4.2, we must consider that PDMPOH_2_^2+^ molecules interact mainly with neutral silanols and with solitary siloxides. The latter are due to deprotonation of the most acidic solitary and well-hydrated silanol moieties. For the latter, in the case of most effective interaction with siloxide *via* dimethylamine, TDDFPT computes emission to be slightly blue shifted: compare Fig. 4E with D. Because of electric interactions with silica anionic sites, effective admixing of PDMPOH_2_^2+^ orbital components with silica electronics shifts the HOMO, LUMO and the next transitions into the near infrared. Since they are of rather low oscillation strengths, they do not provide effective pathways to support emission. As a result, theory suggests that emission happens from a higher energy electronic subset (not from the LUMO): see Fig. 4D and E. We may consider the observed blue shift of PDMPO emission in pH from 3 to 3.6 as a signature of passing the first p*K*_a_ of the silica surface. Accordingly, we ascribe the blue shifted emission to excited 
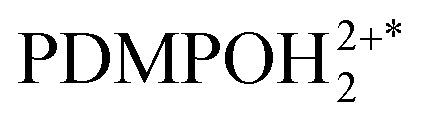
 next to a solitary siloxide. According to the definitions of our model, B_2H_ describes this spectral contribution: it dominates in data measured in experiments involving silica particles in the pH range from 4 to 10: see Fig. 6D. Using this experimental data, we may extract this spectral contribution subtracting a scaled spectrum of PDMPOH^+^ (detected, for example, at pH = 12) from a spectrum detected, for example, for pH = 9, till there is no contribution of the band at 440 nm.

Adopting spectral properties of 
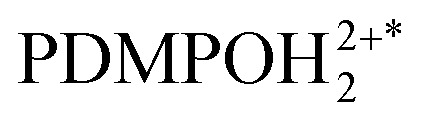
 next to a solitary siloxide, we apply the “aqueous” model without any other changes to compare the computed pH dependent emission as we show in Fig. 6E with the experimental data: see Fig. 6D. To facilitate the comparison, in Fig. 6F we pair selected vertical slices from panel D, which present pH dependent emissions at 442 and 538 nm (blue and red circle lines) with the correspondent modelled dependences (green and black lines) as taken from panel E. For details of the fitting see the ESI.†

Using the “aqueous” model we reproduce spectral properties at very low pH = 2.5, and in the pH range from 10.5 to 14. It is interesting and important that in the pH range from 10.5 to 12, the ratio of the emission bands resembles that for PDMPO chromophores in water. In water such a ratio of bands is conserved in the pH range from 5 to 12. In contrast, application of the “aqueous model” to the results of emission spectroscopy on PDMPO in the presence of silica fails to reproduce the ratio of the two emission bands in the pH range from 3.8 to 10. The difference of the band ratio in this range of pH is obvious in Fig. 6F. It may be used as a numerical measure to show how much the excited PDMPOH^+^* at silica is less accessible to receive a proton from water comparing to the experiment in water (Fig. 6A and C). In Fig. 6G we describe the difference as the access (to proton transfer) function. Technically, we may use this function to replace the *w* parameter. In the “aqueous” model *w* is fixed to describe probability for PDMPOH^+^* to accept a proton, regardless of pH. If instead of a fixed value we adopt the pH dependent accessibility function or its interpolation (as shown in Fig. 6G), we may receive a reasonable agreement with the experimental data, as measured in the presence of silica nanoparticles: this comparison is described in detail in the ESI.†

Beside the anticipated blue shift of the 
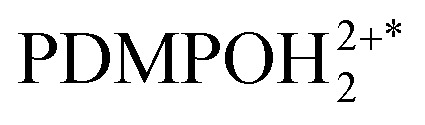
 emission upon the first p*K*_a_ of silica, the extracted PDMPOH^+^* access (to proton transfer) function is another evaluation of the chromophore's sensing capacity of silica surface electronics. This describes the observed switch of the emission band ratio on pH when next to silica. Here, we take advantage of our results of TDDFPT to explain this and to understand possible practical consequences. Again, for pH > p*K*_a1_, observation of the emission band at 530 nm is the signature that in the excited state some of PDMPOH^+^* may receive a proton from water. The relative increase or decrease of such emission (comparing to the band at 460 nm) corresponds to an increase or decrease of the proton transfer probability, which is limited by the lifetime of the excited state and geometry of aqueous moieties proximal to the pyridine nitrogen.

### (6) Molecular mechanisms of PDMPO selectivity to local silica ionicity

Theory suggests that within the pH range from 2 to 3.8, the system contains double protonated chromophores that interact mainly with the oxygen of silanol moieties of a neutral silica surface either directly or *via* water bridges. Such interactions are relatively weak: see Fig. 3. When pH > p*K*_a1_ = 3.8, PDMPOH^+^ becomes the main form in the electronic ground state: binding of it through the dimethylamine group to relatively sparce siloxide moieties are energetically favorable. At the same time, the deprotonated pyridine may interact with water, and with silanol groups either directly or *via* water bridging. Since not ionic, such deprotonated pyridine moieties explore a large intermolecular configuration space. However, when photo-excited under the geometry of water bridging to a hydrated silanol, we may expect very effective proton transfer, due to both, the direct geometry of such water bridging and neutrality of the prevailing silanol moieties. Such a regime may provide the dominance of the emission at 530 nm as conserved in the pH range from 3.8 to 10: see Fig. 6D.

At this point, it is important to relate theory estimations for electronic responses of single protonated PDMPOH^+^ next to a silica surface, when it becomes more polar after passing its second deprotonation. When there is a sufficient surface density of siloxides, beside polar binding of dimethylamine groups to a silica anionic site, deprotonated pyridine moieties may water bridge with a siloxide, as well: data in Fig. 3 indicates a larger energy binding for such systems. However, under such geometries the very proximity of the negatively charged siloxide imposes an electric force to prevent a proton transfer *via* the bridging water and from any neighboring aqueous cluster to the nitrogen of the pyridine moiety. When this is the case, a proton transfer would require a detachment and a reorientation of the pyridine moiety to search for a suitable aqueous donor. This is analogous to the situation of PDMPOH^+^* in water. Therefore, when a sufficient density of siloxides exists (this may correspond to the second silica deprotonation p*K*_a_), the increasing population of PDMPO(H^+^) to associate with one siloxide *via* its dimethylamine and with another siloxide *via* water bridging to pyridine may account for the observed relative increase of the emission band at 430 nm, which we relate with a smaller capacity of excited PDMPOH^+^* to except a proton. From this perspective, the intermediate point of the access function (Fig. 6G) at about pH = 9 may correlate with the second deprotonation p*K*_a_ of the silica nanoparticles used in the experiment.

### (7) Practical implications: 3D ionic tomography *via* confocal microscopy

The suggested description of the mechanics of PDMPO's sensing capacity in respect to silica surface electronics are valuable in microscopy applications. While discussion on the accessibility function (Fig. 6G) to explain the emission bands ratio change concerns the average charge of the silica surface of all silica nanoparticles in a bulk sample, an experiment under microscopy resolution draws attention to the role of spatial distribution. Understanding the dynamics of material and information exchange with environment for organisms with silica skeletal elements to sustain healthy biochemistry is also relevant for the engineering of mineral implants to support healthy physiology on the microscale. In this respect, efforts^76^ to control biosilica properties in diatoms is an example of an emerging playground where *in vivo* detection of how microscale silica ionic character may correlate with physiology.

Up till now, recent microscopy experiments to generate and utilize silica nanoparticle zeta potential calibration^40^ using microscopy sampling of PDMPO emission band ratio while scanning bioinorganic silica provides the most handy and straightforward approach to anticipate variance of local ionicity. Results of the presented analysis suggests a possibility to relax the dependence on an external calibration, and to specify the second deprotonation p*K*_a_ of silica locally while adopting microfluidic microscopy to sample the band ratio (accessibility functions) under pH = 7, 9 and 11. Correlating this with structural properties may be the necessary prerequisite to plan differential emission microscopy to sample living matter biosilica interactions upon external perturbation – the tasks specific to physiology and toxicology of living matter. Importantly, as TDDFPT suggests for PDMPO–silica associates that HOMO to LUMO transitions shift into the near infrared spectral range, the possibility to detect emission from such transitions, in the region of tissue transparency, may offer *in vivo* diagnostic capacity to sample electronic properties of silica-containing implants.

**Fig. 7:**
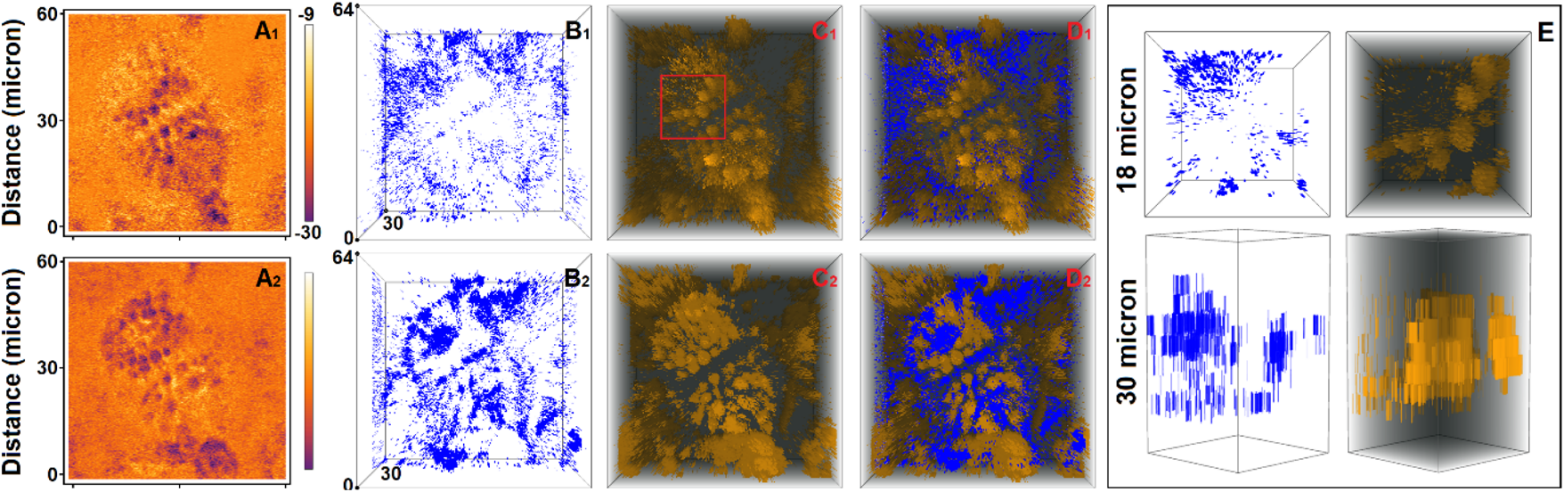
Confocal microscopy of stomata ionic states. (A) Ionic map of a selected layer, (B) single color visualisation of tomographic assembly of sites with ionicity higher than −14. (C) Single color shadow enhanced visualisation of tomographic assembly of sites with ionicity lower than −24. (D) Combination of tomographic presentations as shown in panels B and C. Subscript indices 1 and 2 indicate results received using single-color and two-color experimental setting. In the first case we excite at 405 nm to collect images within 460–470 nm, and within 500–510 nm. In the second case we use image detected within 460–470 nm using 405 nm excitation, and image detected within 609–750 nm using 532 nm excitation. (E) Zoom in of the tomographic assemblies as in B_1_ and C_1_ (see the red box in C_1_) presented from the top (upper set) and from the side (lower set).

As an example, we explore the sensing capacity of PDMPO to address the structure and physiology of *Equisetum arvense* stomata. Specifically, in Fig. 7A_1_ and A_2_ we present ionic maps computed using single-color and two-color excitation experiments for the layer (in respect to vertical *Z* axis) of the highest fluorescence intensity according to the ionic mapping protocol as we described previously.^39^ Again, since DFT suggests rather low energy of the HOMO–LUMO transition for PDMPO next to silica, observation of its emission in the spectral ranges 460–470 nm and 600–750 nm is possible as energy relaxation may involve mechanisms that compromise Kasha's rule.^69^ Both maps agree on the low ionicity of silica knobs (darker spots) next to the aperture edges of the subsidiary cells and relatively higher (light yellowish region) ionicity along the teeth of the aperture margins. The two maps slightly disagree on the ionicity of the stomata regions sampled next to its borders. Overall, the two-color image is slightly crisper. This is more obvious if we sort contributions of sites with ionicity higher than −14 from every sampled layer to plot (as blue points) in 3D: see Fig. 7B_1_ and B_2_. We may note that the high ionic sites in Fig. 7B_1_ have a more diffuse distribution in space. Instead, the high ionic sites in Fig. 7B_2_ contrast better the border line of the stomata, the aperture and the knob structures. To make this more obvious, first, in Fig. 7C_1_ and C_2_ we sort contributions of sites with ionicity lower than −14 from every sampled layer to plot (as yellow points) in 3D; and, second, we superimpose 3D contributions of the sites of high and low ionicity in Fig. 7D_1_ and D_2_. Slightly lower contrast of the ionic map extracted using the single-color excitation experiment may suggest either a tiny contribution of PDMPOH^+^* emission in the spectral window 500–510 nm, or that excitation at 405 nm may stimulate proton dynamics between 
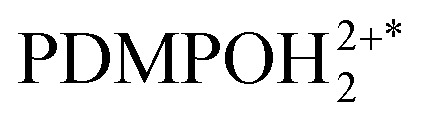
 and silica. From this perspective, adopting image detection within the 600–750 nm range while using 532 nm excitation may suggest a better-quality sampling of the 
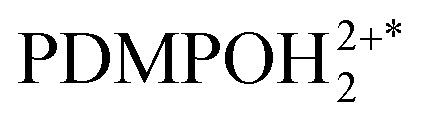
 contribution. At the same time, such an experiment is significantly more demanding due to the lower yield of the emission.

In Fig. 7E we present an expanded view of the 3D ionic tomography of high and low ionic states in the spatial region of stomata aperture, as indicated with the red box in Fig. 7C_1_. The distribution of low ionic states (yellow) as shown from the side (right image in the lower set) reveals that, silica depositions, which start at the surface as knob-like structures, continue either as pillars along the cell side or as pillars that protrude into the structure as far as 30 microns in depth. It is interesting that the high ionic states (blue colored) tend to cluster in the complementary neighboring spaces systematically: comparing the left and right images in the lower set in Fig. 7E, we may note that higher ionic space fills the slightly open aperture and the chamber below.

**Fig. 8:**
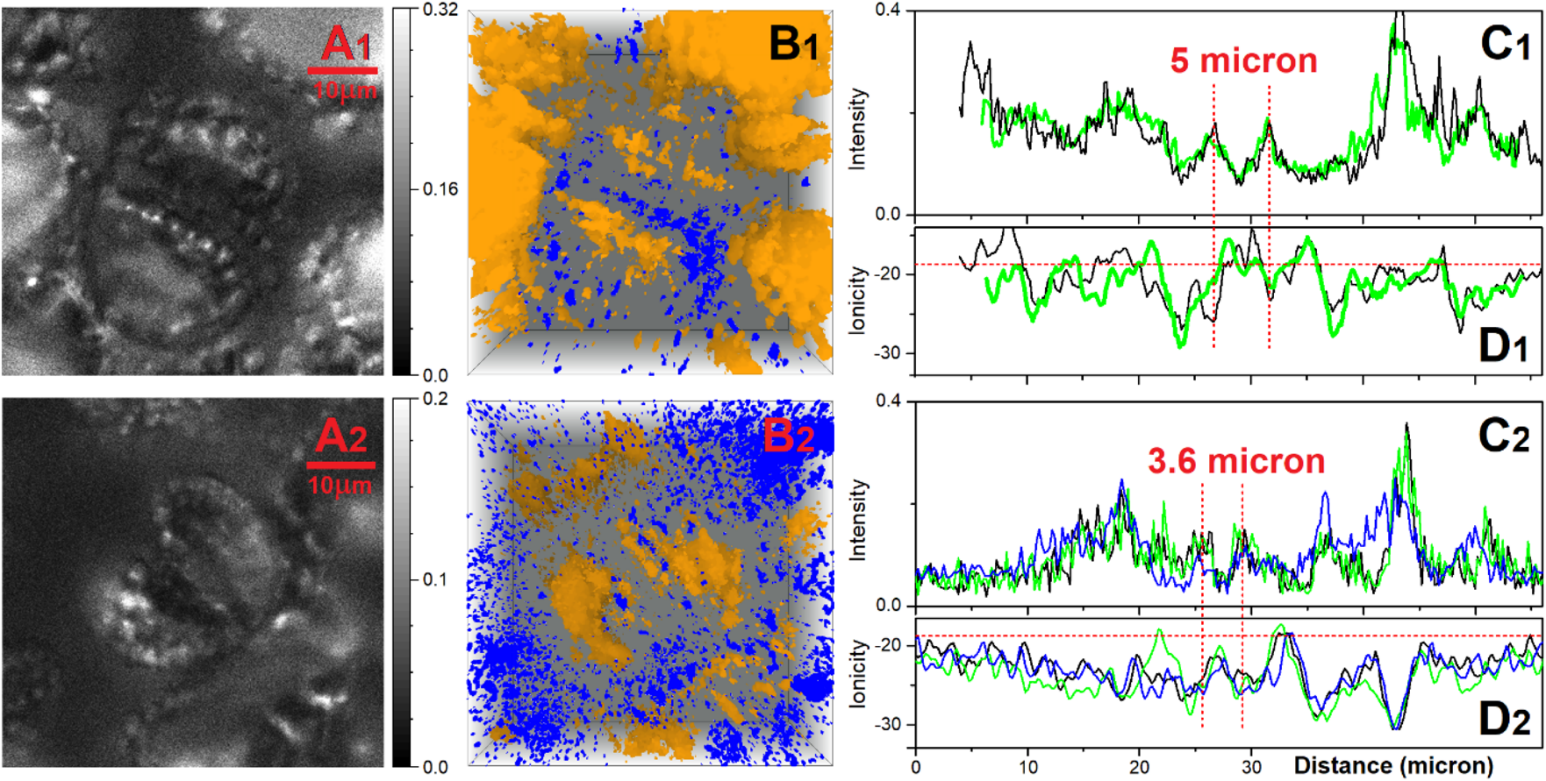
Confocal microscopy of ionic states of open (upper panels labelled with subscript 1) and closed stomata (lower panels labelled with subscript 2). (A) Emission image of a selected layer detected within 500–510 nm using excitation at 405 nm. (B) Tomographic superposition of lower (yellow) and higher (blue) ionic states, as in Fig. 7D. (C) Emission sampled across the stomata aperture using images as in panel A but for several layers (black, green, blue lines). (D) Ionicity sampled across the stomata aperture for several layers of ionic maps (black, green, blue lines).

Following these observations, we conducted tomography of ionic states for a number of stomata (open and closed) and in Fig. 8 correlations of selected slices across emission images and ionic images of the aperture of stomata where edges are separated by 6 and 3.6 micron (open and closed) to sustain or not, respectively, a density of relatively high ionic states in the opening.

## Discussion

Identification of the local ionic character of biosilica at microscopic resolution is of particular importance – as it may help increase our understanding of the mechanisms of growth and locomotion, as well as biochemistry of responses specific to organisms, which rely on inclusions of silica structural components.^1–6^ Borrowing from nature one may search for inspiration to design silica based functional implants and sensors. Considering the diversity and complexity of silica interfaces at the microscale,^15,76–78^ characterization of ionic states at mineral and biomineral interfaces at sub-micron resolution and under physiological conditions is an important requirement for effective micro and nano engineering. In this respect, progress in the microscopy of PDMPO at bio-interfaces^40^ offers a promising opportunity to resolve differently charged domains at the surface of silica with discretion down to half the wavelength of detected light: moreover, two-photon excitation of PDMPO emission in the spectral range of tissue optical transparency may allow *in vivo* ionic diagnostics of implants with silica inclusions.

The ionic tomography of *Equisetum* stomata we present in Fig. 7 and 8 demonstrates how 3D sampling of low ionic sites reveals that silica depositions in the knobs (on the epidermis) persist as 30 microns pillars below the surface. We may hypothesize that, originally, the pillars were the vessels to the pores or ectodesmata to deliver silicic acid,^24^ and, later, started accumulating cutin or cellulose and callose. Earlier studies reported that, possibly, silica is present intercalating depositions between the fibrillar cellulose of the epidermis cell walls to provide the characteristic ribs composed of cellulose impregnated with silica.^24,25^ Here, according to 3D ionic tomography we suggest that beside the noticed ribs at the surface, silica impregnated cellulose pillars protrude inside the cells. The bioinorganic components give rise to a structured *exo* and *endo*-skeleton, the strength and flexibility of which is according to an interplay between the mineral and organic contributions.

To provide both, mobility and rigidity is the challenge *Equisetum* has solved for stomata functionality developing its *exo* and *endo*-skeletal components. Previous studies indicated that biomineralization may compromise movements in older stomata.^25,30^ This, however, may not contradict the main role of stomata – to provide diffusion of carbon dioxide and water.^17^ In the light of the geological record on the relative presence of methane, oxygen and carbon dioxide,^79^ here, we note that moderation of CO_2_ consumption could be the main task of biosilicified stomata. Moreover, within the last two decades, efficiency of porous silica to retain CO_2_ while in a mixture with methane (CH_4_) has been characterised experimentally and theoretically to develop industrial applications.^80–84^ As an example, recent molecular dynamics simulations for OH and methyl (CH_3_) terminated silica nanopores indicated higher adsorption of CO_2_ compared to methane (CH_4_) with higher diffusion coefficients for CH_4_ compared to CO_2_.^85^

From this perspective, a large variance of ionicity upon stomata aperture opening may present a palette of surface silica states to moderate capturing CO_2_ at the necessary level depending on the environmental conditions. According to the third law of thermodynamics, matter tends to distribute uniformly. High contrast ionic gradients upon stomata aperture opening may present a paleo-example of the biological control of local entropy.

## Conclusions

To interpret the spatial distributions of optical states in terms of local chemistry we compare experimental spectral signatures with those computed using quantum theory. To tackle this for PDMPO chromophores when next to silica of different ionic charge, here, we adopt *ab initio*, DFT and TDDFPT to (a) model structural possibilities, (b) describe the electronic states and their perturbations upon interactions, and (c) express optical properties which we relate to those detected experimentally.

Using computed properties, we model PDMPO emission dependence on pH, in water and in the presence of silica, and describe structural and electronic variations showing the sensitivity of the PDMPO chromophore to the ionic diversity of silica interfaces. The experiment may also be extended to two-photon microscopy in the spectral region of biological tissue optical transparency, where emission may help address the local biochemistry and physiology of implanted silica containing micro-devices.

Comparing computed and experimental electronic spectra (absorption and emission), to test the selectivity of PDMPOH^+^* and 
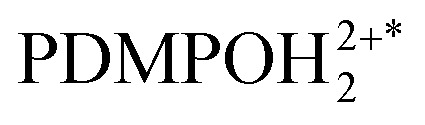
, we extend emission sampling to the near infrared for confocal microscopy under single-colour and two-colour excitation regimes. Using both approaches we demonstrate 3D ionic tomography of *Equisetum* stomatal complexes with ionicity differences under sub-micron resolution. In these specific samples, 3D sampling enabled the discovery that silica depositions, which start at the surface as knob-like structures at the epidermis, continue either as pillars along the cell side or as pillars to protrude from subsidiary cells inside the structure as far as 30 microns, as an example of an *exo*-/*endo*-skeleton.

Considering the needed mobility of the stomata, we suggest that the necessary flexibility of the bioinorganic *exo*-/*endo*-skeleton is due to the character of the composite structure: silica depositions intercalate between the fibrillar (likely cellulose) scaffold of a pillar. Using three-dimensional ionic tomography allowed us to distinguish that upon stomatal opening, the aperture accommodates a density of relatively high ionic silica states, while the very proximal knobs of the aperture margins are of rather low ionicity. Considering that during the time of Equisetaceae origin and proliferation (pre-Silurian to Mesozoic) the geological record indicates significant variation in carbon dioxide, methane and oxygen levels,^79^ we propose the high contrast of ionicity upon stomata opening is to moderate uptake of carbon dioxide (necessary for optimal carbon gain) under various atmospheric conditions. This we consider to be an example of bioinorganic molecular mechanochemistry to modulate local entropy for favourable transport. In support of this suggestion, during the past two decades numerous experimental and theoretical studies have been performed to show the high efficiency of silica nanopores in controlling diffusion coefficients for CH_4_ and CO_2_ molecules, with dependence on the chemistry of silica surfaces.^85^

## Data availability

The data supporting this article have been included as part of the ESI.†

## Author contributions

VVV: conceptualization, investigation, formal analysis, data curation, methodology, visualization, writing- original draft, review and editing; GJH: methodology, investigation, writing-review and editing; CCP: conceptualization, investigation, formal analysis, funding acquisition, writing-original draft, review and editing.

## Conflicts of interest

There are no conflicts of interest to declare.

## Supplementary Material

SC-016-D4SC07973F-s001
