# Supplementary material for: Stomata biosilica and Equisetum photosynthesis: ionic tomography insight using a PDMPO silicaphilic probe
Source: Chem Sci. 2025 Apr 24;16(21):9509–24. doi: 10.1039/d4sc07973f (PMC12042103; doi:10.1039/d4sc07973f)
Supplement: SC-016-D4SC07973F-s001 [file SC-016-D4SC07973F-s001.pdf]

Supplementary Information (SI) for Chemical Science.
This journal is © The Royal Society of Chemistry 2025

Supplementary materials

Stomata biosilica and equisetum photosynthesis: ionic tomography insight using PDMPO silicaphilic probe

*Victor V. Volkov, Graham J. Hickman, and Carole C. Perry**

Interdisciplinary Biomedical Research Centre, School of Science and Technology, Nottingham Trent University, Clifton Lane, Nottingham, United Kingdom NG11 8NS

Figure S1 presents images of structures of single and double protonated PDMPO chromophore as optimized in vacuum using density functional theory.

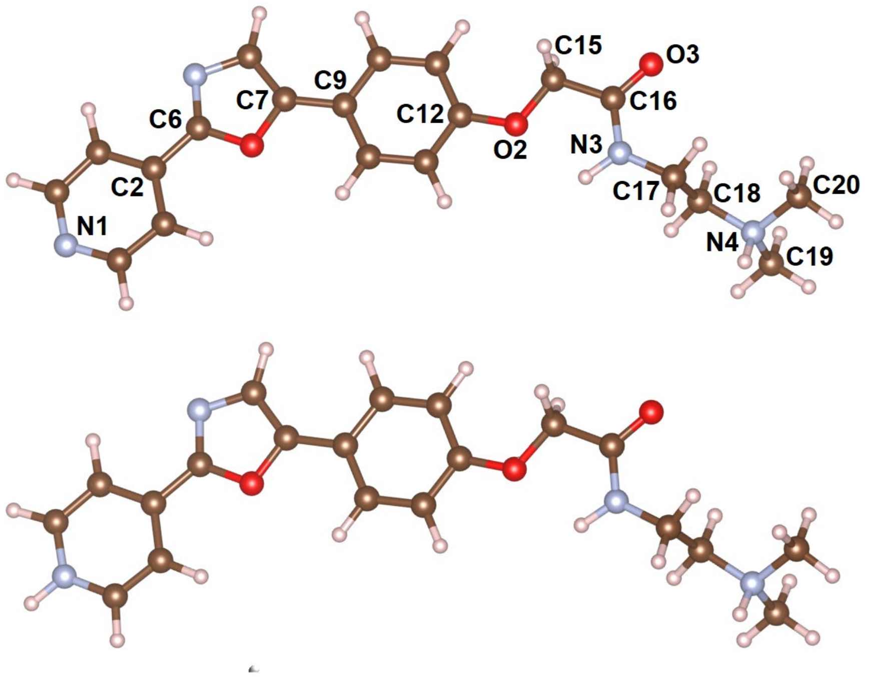

Figure S1. *Structure of PDMPOH$^+$ (top) and PDMPOH$_2^{2+}$ (bottom).*

To address the role of charge transfer transition in optical excitations [1], and to distinguish contribution of vibronic envelope in spectral broadening, we employ the 6-31++g(d,p) basis set under B3LYP exchange-correlation functional [2] as implemented in the Gaussian 16 program [3]. Specifically, using "Pop=DCT" under TD-DFT we compute several of the ground to excited state electronic transitions.

For PDMPOH$_1^{1+}$ we obtain:

State	w(eV)	R+(Ang)			R-(Ang)			D(Ang)	QP(a.u.)	QP+QM
		x	y	z	x	y	z			
1	3.380	-6.379	0.700	0.006	-1.552	-1.252	0.118	5.208	0.630	1.1D-07
2	4.349	-1.647	-1.015	0.069	-2.491	-0.933	0.111	0.849	0.429	3.9D-06
3	4.381	-5.825	0.339	0.049	-8.465	2.434	-0.239	3.383	0.777	-2.3D-07

For PDMPOH$_2^{2+}$ we obtain:

State	w(eV)	R+(Ang)			R-(Ang)			D(Ang)	QP(a.u.)	QP+QM
		x	y	z	x	y	z			
1	2.698	-6.803	0.924	-0.021	-1.202	-1.127	0.075	5.965	0.740	3.6D-07
2	3.821	-6.376	0.678	0.005	-0.461	-0.811	-0.031	6.099	0.899	-7.3D-07
3	3.914	-7.374	1.502	-0.107	-2.143	-1.027	0.111	5.815	0.880	-3.2D-06

Here,
"R+(Ang)" columns x,y,z are the Cartesian coordinates of the centroid of the e-dens. increase region;
"R-(Ang)" columns x,y,z are the Cartesian coordinates of the centroid of the e-dens. depletion region.
"D(Ang)" column is the distance (in Angstrom) between the centroids.
"QP(a.u.)" is the amount of charge (in electrons) depleted upon excitation.
"QP+QM" is simply a check for the charge integration quality: "QP+QM" should be zero.

As one may see, for PDMPOH$^+$, upon the first transition there is a separation of 5.208 Angstroms between the centres of density depletion and density increase regions for a total of 0.630 electron charge depletion/increase; for PDMPOH$_2^{2+}$, upon the first transition there is a separation of 5.965 Angstroms between the centres of density depletion and density increase regions for a total of 0.7400 electron charge depletion/increase. In both cases, the charge transfer is less than one electron. This suggests that there are overlaps between the depletion and increase regions. However, with a distance of over 5 and 6 Angstrom between the centres the induced charge separations are significant, particularly in the case of PDMPOH$_2^{2+}$.

Next in Figure S2, we present TDDFT studies of optical absorption and emission spectra of PDMPOH$^+$ and PDMPOH$_2^{2+}$ while accounting for the vibronic envelope in Franck-Condon region.

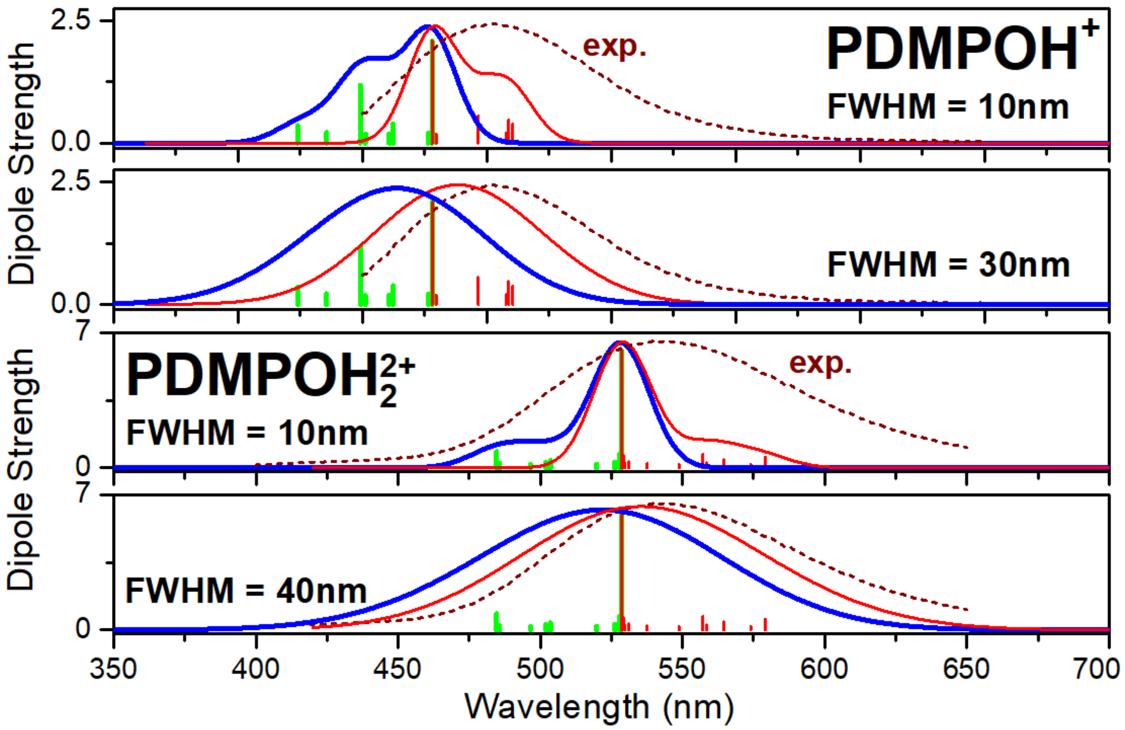

Figure S2. *Absorption (blue line) and Emission (red line) spectra computed for PDMPOH$^+$ and PDMPOH$_2^2$. Green and red vertical lines indicate resonances and Dipole Strength values for the absorption and emission vibronic transitions, respectively. Dashed dark red lines present experimental fluorescence data according to Ref. 4. We express spectra taking convolutions while adopting Gaussian line-shapes with widths as indicated.*

As one may see, the computed spectra suggest that asymmetry of the experimental spectra as reported earlier [4] may be assigned to vibronic contributions.

Next, using the Tight Binding approach [5], we address dynamics in vacuum, water and at a hydrated silica monolayer according to the geometry of α-cristobalite [6].

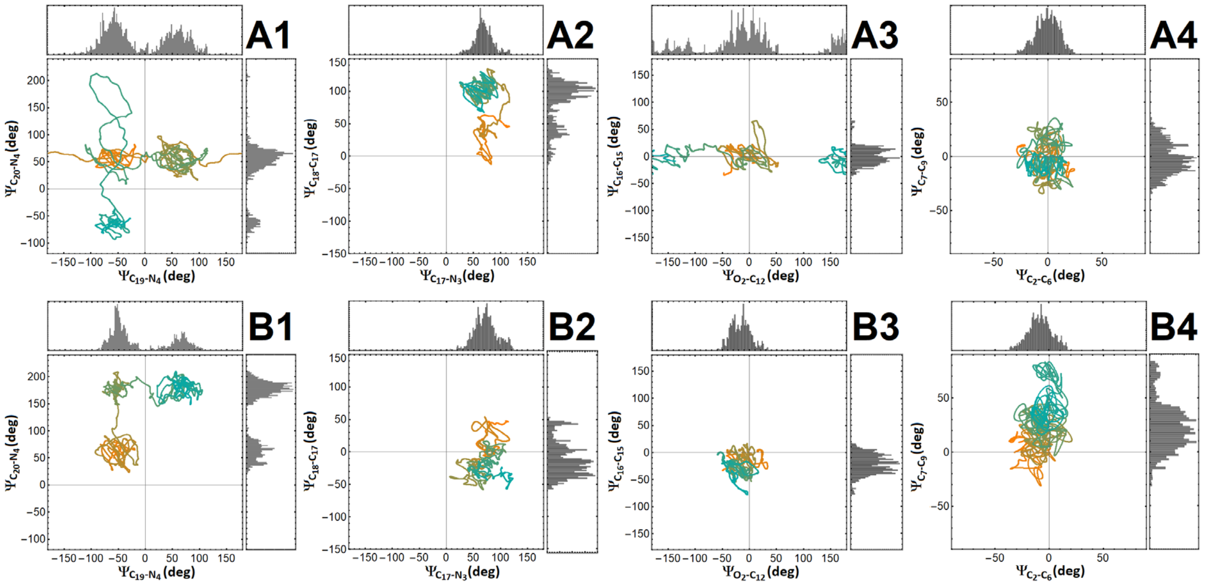

Figure S3. *Dihedral angles, as shown in each panel, about a bond according to the atomic labeling in Figure S1 extracted along dynamic trajectories simulates for PDMPOH$^+$ chromophore in vacuum (A1-A4) and in water (B1-B4). We use a color blend from Orange to Dark Cyan to depict the properties as sampled from the start to the end of the simulated trajectory, respectively.*

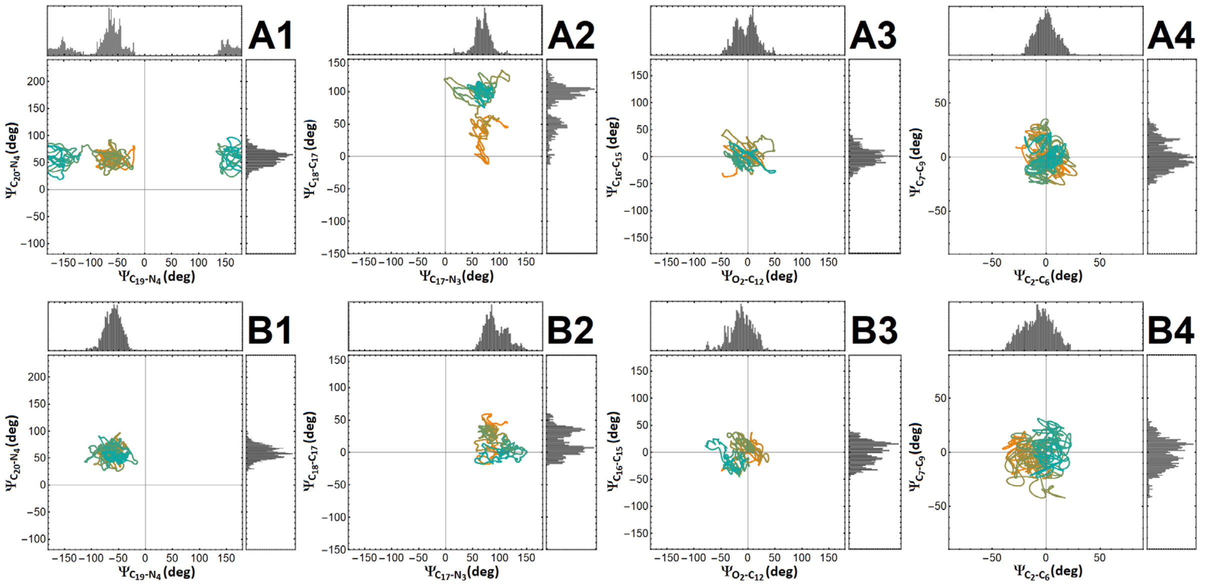

Figure S4. *Dihedral angles, as shown in each panel, about a bond according to the atomic labeling in Figure S1 extracted along dynamic trajectories simulated for PDMPOH$_2^{2+}$ chromophore in vacuum (A1-A4) and in water (B1-B4). We use a color blend from Orange to Dark Cyan to depict the properties as sampled from the start to the end of the simulated trajectory, respectively.*

The geometry of the aromatic moiety is determined by the torsion angles about the C2-C6, C7-C9 and O2-C12 bonds (Figure S1).

Both, in vacuum (Figure S3: panels A3, A4, B3, and B4) and in water (Figure S4: panels A3, A4, B3, and B4), the angles fluctuate (in the range $\pm 50^o$) about 0° to provide a more or less flat geometry of the aromatic group.

Additionally, for the most of the chromophore configuration space, rotation dynamics about O2-C12, C15-C16, C13-N3, and C17-C18 bonds define structure of the of aminoethyl-aminocarbamoyl-methoxy component. In vacuum and in water, the angles about O2-C12 and C15-C16 bonds tend to vary around 0° (or 180°).

However, these may demonstrate π flips around the bonds: for examples, see Figures S3A3 and S6D3. When a π flip occurs about the C15-C16 bond that carbonyl atom O3 points toward the ether O2 atom, for such conformer DFT predicts a higher energy.

Therefore, in Figure S1 we image the lower energy structures. Furthermore, we give preference to such conformers preparing initial structure to simulate properties when next to silica.

When next to silica, distributions of the angles demonstrate broadenings and asymmetry in distributions of numeric values about the means (for examples, see Figure S5: panels A3, A4, B4, D3).

The data indicate departure of the aromatic moiety from a flat geometry and asymmetry of interactions of the distorted structure that are induced upon association with the surface.

When in vacuum and in water, fluctuations of the rotation angles (about O2-C12, C15-C16, C13-N3, and C17-C18 bonds) do not show obvious dependences on each other, and their distribution functions (one-dimension projections) are homogenous, overall.

However, when at silica, rotation dynamics about O2-C12 and C15-C16 bonds demonstrate anticorrelation character (for example, in case of $PDMPOH^+$, see Figure S5: panels A3, B3, D3, and D4).

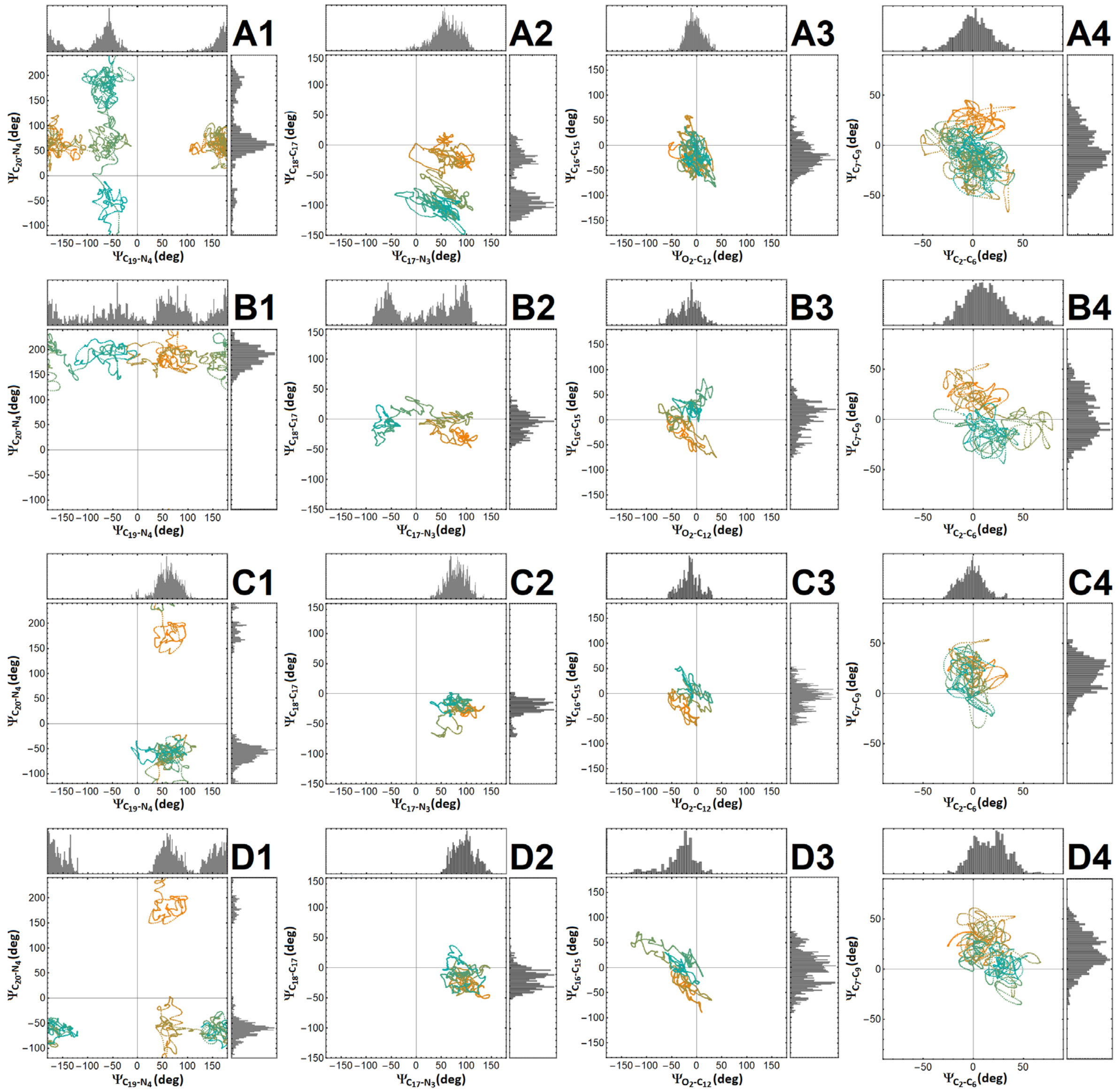

Figure S5. *Dihedral angles, as shown in each panel, about a bond according to the atomic labeling in Figure S1 extracted along dynamic trajectories simulated for PDMPOH$^+$ chromophore when next to a well hydrated silica monolayer without siloxide moieties (A1-A4), with a single siloxide group proximal to the head of the chromophore (B1-B4), with two siloxide sites proximal to the head and the tail of the chromophore (C1-C4), and with a single siloxide moiety next to the tail of the chromophore (D1-D4). We use a color blend from Orange to Dark Cyan to depict the properties as sampled from the start to the end of the simulated trajectory, respectively.*

Overall, when next to silica, the configuration space of the dimethyl- aminoethyl-aminocarbamoyl-methoxy component becomes larger due to anisotropy specific to the surface. Departure of all structural components from planarity as computed in vacuum and in water is one of the manifestations of numerous local energy minima. Another manifestation of the wider configuration space is the enhanced heterogeneity and asymmetry in distributions of angular correlations (for examples, see Figures S5: A2, B2, A1, C4). This is because when interactions with the surface distorts the bilateral structure, intermolecular arrangements (with water and silica) are different on the two side of chromophore.

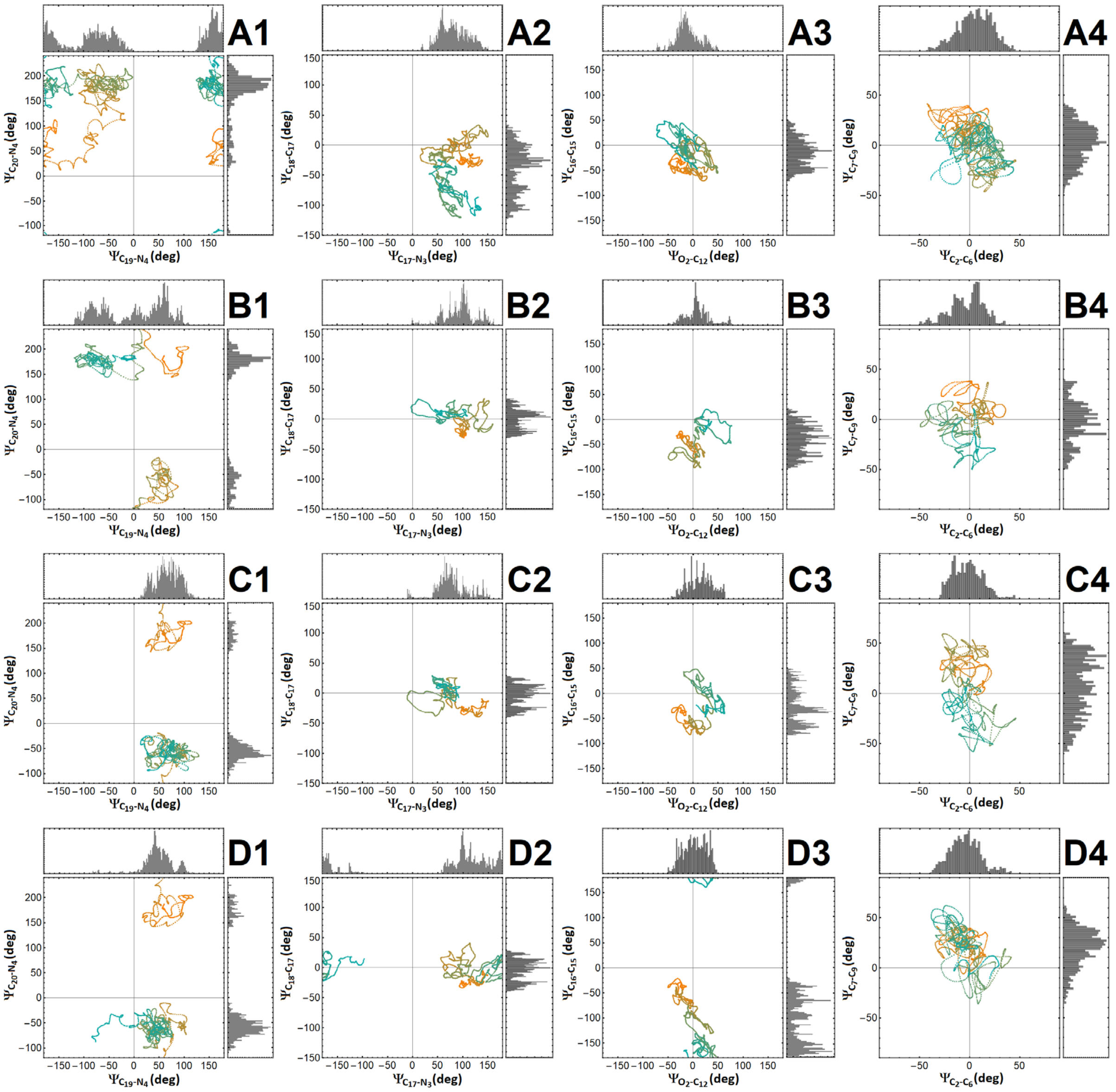

Figure S6. *Dihedral angles, as shown in each panel, about a bond according to the atomic labeling in Figure S1 extracted along dynamic trajectories simulated for PDMPOH$_2^{2+}$ chromophore when next to a well hydrated silica monolayer without siloxide moieties (A1-A4), with a single siloxide group proximal to the head of the chromophore (B1-B4), with two siloxide sites proximal to the head and the tail of the chromophore (C1-C4), and with a single siloxide moiety next to the tail of the chromophore (D1-D4). We use a color blend from Orange to Dark Cyan to depict the properties as sampled from the start to the end of the simulated trajectory, respectively.*

Next in Figure S7 we provide extended review of Hydrogen bond dynamics for PDMPO chromophores under different protonation at the considered silica monolayers.

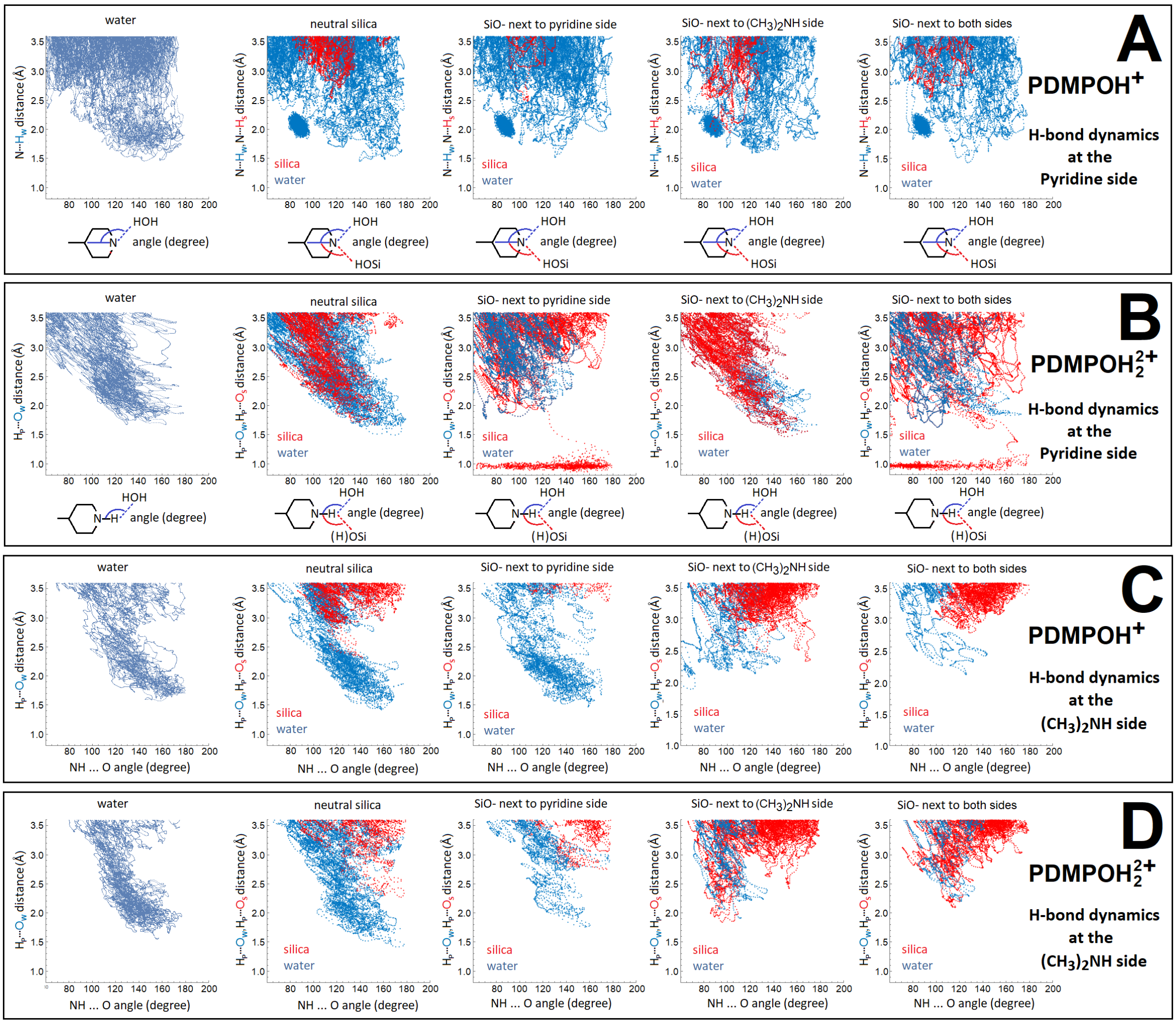

Figure S7. *Extended review of Hydrogen bond dynamics predicted by Tight Binding molecular dynamics.* ***A:*** *characteristics of hydrogen bond dynamics to involve pyridine moiety of PDMPOH$^+$ chromophore when in water, next to hydrated silica S0, silica S1 with siloxide moiety proximal to pyridine side, silica S1 with siloxide moiety proximal to dimethyleamine, and silica S2 with two siloxide groups proximal to both sides of the PDMPO (from left to right, as indicated). Blue and red dotted lines present geometric properties specific to hydrogen bond dynamics upon interaction with water and silica, respectively. Subscripted letters W and S in axes labels indicate atoms of water and silica, respectively.* ***B:*** *characteristics of hydrogen bond dynamics to involve pyridine moiety of PDMPOH$_2^{2+}$ chromophore for the systems as described for Panel A. Here, since the pyridine moiety is protonated, the atoms to measure angles and distances are different: see the axes labels. Subscripted letter P in axes labels indicate atoms of pyridine.* ***C:*** *properties of hydrogen bond dynamics to involve dimethyleamine moiety of PDMPOH$^+$ chromophore for the systems as described for Panel A.* ***D:*** *properties of hydrogen bond dynamics to involve dimethyleamine moiety of PDMPOH$_2^{2+}$ chromophore for the systems as described for Panel A.*

Information presented in Figures S5, S6 and S7 suggest sixteen initial structural arrangements to refine structural properties. First, considering that experiments are conducted at thick silica, we extend the systems to two-layer silica structures adding a second silica layer below the original monolayer, according to the geometry of α-cristobalite [6,7]. Second, we conduct quantum *ab initio* Born-Oppenheimer molecular dynamics (BOMD) under thermalization regime followed by a short trajectory

to sample conformational averages about the received mineral structure. Third, we conduct structural optimizations using DFT.

Sampling various PDMPO structural realizations at silica, we would like to evaluate, approximately, how strong is the energy gain for PDMPO to associate with silica under different protonation conditions and what happens to the electronic properties of PDMPO and silica when they interact. Such information would characterize (overall) how strong PDMPO silicaphilic tendencies are in dependence on its protonation and conditions at the surface.

To manage this, we conduct additional series of theoretical studies. Specifically, we review each structural case of interest, remove water molecules which do not participate in bridging between the PDMPO chromophore and silica, and conduct structural optimizations for such nearly completely dry versions of the systems. Consequently, for each optimized case, we compute energy for silica (after we remove PDMPO atoms) and PDMPO (after removing silica and associating water with it, to maintain its bridging contribution). In Figure S8, for each case, we image interacting moieties and list the corresponding binding energies. Here, it is important to stress, that in order to evaluate interaction energy between chromophore and silica, we conduct binding studies at essentially dry interfaces: for each structural case, we remove interfering water and reoptimize structure. Therefore, the binding energies, for the initial hydrated cases, are approximate. They are only to compare tendencies for the cases. We may see that neutral silica is somewhat attractive to both protonated charged forms of PDMPO. Of course, binding energy increases with silica ionicity.

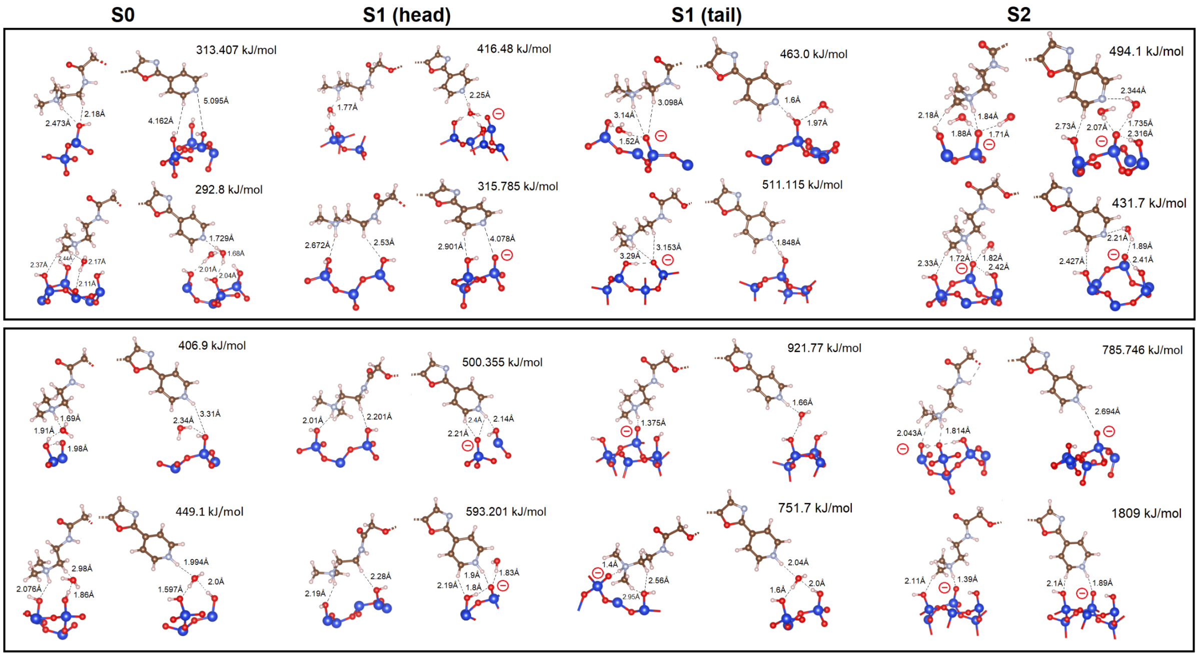

Figure S8. *Energetics of binding for selected cases of PDMPOH^{+} (top) and PDMPOH$_2^{2+}$ (bottom).*

Next, we return to relatively hydrated versions of the systems to sort out twelve representative cases for time-dependent DFT to evaluate optical electronic properties. In Tables S1 and S2 we list dihedral angles of the chromophores in such systems to compare with the corresponding values when molecules are optimized in vacuum.

Table S1. *Structural properties of PDMPOH⁺ chromophore after DFT optimization of structures selected from MD simulations when in vacuum, and next to a wet double layer of silica when it is neutral (S0), with one (S1) and two (S2) siloxide moieties. Dihedral angles and their notations correspond to those, as shown in Figures 1, 3-6. Angles about the C20-N4 and C20-N4 bonds are evaluated adopting comparative Hydrogen atoms in methyl groups.*

structure	$\psi(C_{20}-N_4)$ Deg.	$\psi(C_{19}-N_4)$ Deg.	$\psi(C_{18}-C_{17})$ Deg.	$\psi(C_{17}-N_3)$ Deg.	$\psi(C_{16}-C_{15})$ Deg.	$\psi(O_2-C_{12})$ Deg.	$\psi(C_7-C_9)$ Deg.	$\psi(C_2-C_6)$ Deg.	E_{bind} (kJ/mol)
vacuum	177.6088	178.9487	173.4934	94.2398	0.7883	179.2541	0.6302	0.7817	
S0: str.1	177.8874	164.3143	165.1077	118.8158	22.3205	178.6728	7.5951	6.6207	292.8
S1: str. 2	66.3236	63.0621	173.5752	91.2837	2.7951	164.5097	6.8809	10.7853	463.0
S2: str.3	177.3720	52.2258	164.2451	133.6405	0.4426	170.4292	12.6045	12.1107	494.1

Table S2. *Structural properties of $PDMPOH_2^{2+}$ chromophore after DFT optimization of structures selected from MD simulations when in vacuum, and next to a wet double layer of silica when it is neutral (S0), with one (S1) and two (S2) siloxide moieties. Dihedral angles and their notations correspond to those, as shown in Figures 1, 3-6. Angles about the C20-N4 and C20-N4 bonds are evaluated adopting comparative Hydrogen atoms in methyl groups.*

structure	$\psi(C_{20}-N_4)$ Deg.	$\psi(C_{19}-N_4)$ Deg.	$\psi(C_{18}-C_{17})$ Deg.	$\psi(C_{17}-N_3)$ Deg.	$\psi(C_{16}-C_{15})$ Deg.	$\psi(O_2-C_{12})$ Deg.	$\psi(C_7-C_9)$ Deg.	$\psi(C_2-C_6)$ Deg.	E_{bind} (kJ/mol)
vacuum	177.7788	61.5089	173.4951	93.9894	1.2639	179.4790	0.7558	1.2205	
S0: str.1	172.1532	58.9729	161.8394	141.1798	8.6688	168.6718	9.0300	7.0338(	406.9
S0: str.2	177.7593(	46.5427	154.1995	119.4877	7.4612	167.3698	6.3420	9.3096	422.1
S0: str.3	174.6907	60.3886	159.0611	178.9005	4.6620	169.4248	0.1488	3.7001	449.1
S1: str.4	171.9888	61.0204	165.3793	157.1986	5.7518	171.5637	10.1732	4.8483	528.2
S1: str.5	176.1827	58.5254	155.3191	123.2920	2.8889	169.117	6.8523	9.4591	576.2
S1: str.6	167.0202	51.7985	164.3596	176.6992	4.3966	168.6213	0.8006	3.2205	751.7
S2: str.7	174.3657	58.5091	168.3662	151.3501	2.6910	166.4295	14.3436	10.9662	712.6
S2: str.8	177.9206	57.0893	159.1716	177.0083	5.4544	167.0003	8.4332	9.253	847.0
S2: str.9	169.0266	52.7028	166.2798	128.0832	2.7547	169.78	3.6867	8.8913	926.9

Figure S9 presents computed optical absorption resonances.

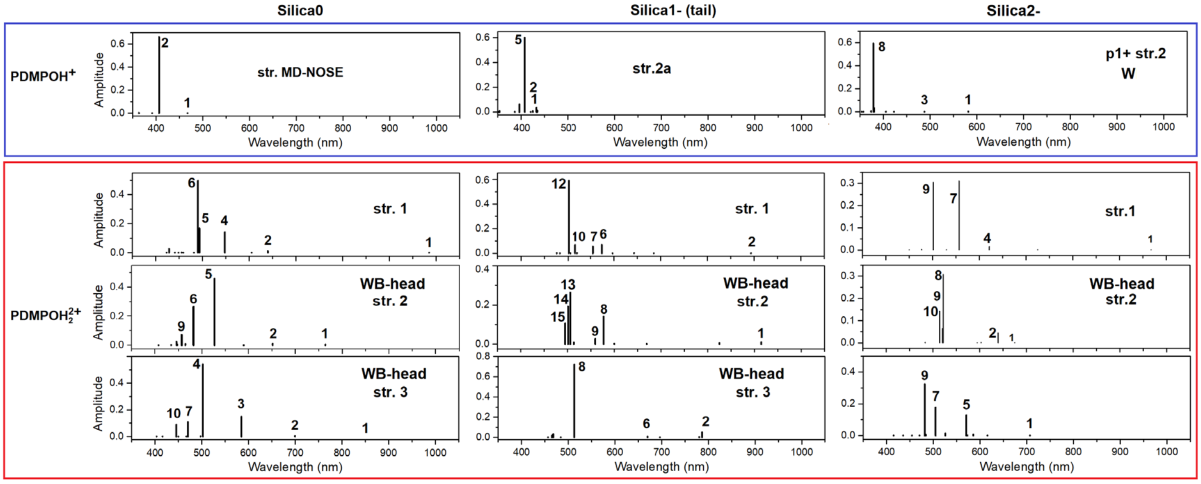

Figure S9. *Optical absorption resonances computed for the selected structural cases using time-dependent DFT.*

Emission in dependence on pH

In Figure S10 (in panel D) we present PDMPO emission dependence on pH while in water. Here, we have to develop a model to approach fitting the dependence.

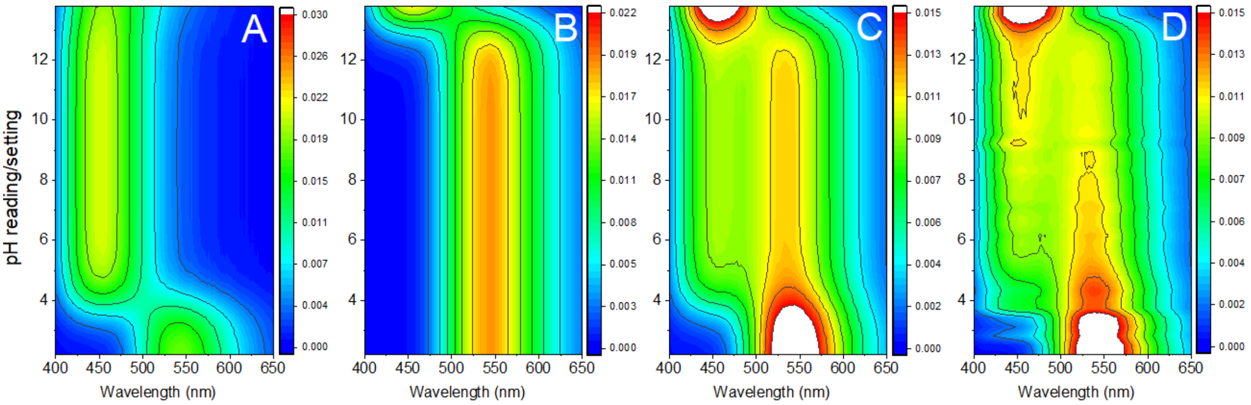

Figure S10. *Modelling of PDMPO emission dependence on pH while in water.* ***A****: computed emission of PDMPO which does not experience proton transfer in electronic excited state.* ***B****: computed emission of PDMPO which does experience proton transfer in electronic excited state.* ***C****: computed total emission.* ***D****: experimental data where each spectrum is normalized on its sum intensity.*

1) Concentrations of PDMPO protonation states in electronic ground state in dependence on pH.

According to equation for the first protonation in the ground state:

$$\mathrm{PDMPO} + \mathrm{H}^{+} = \mathrm{PDMPOH}^{+} \qquad (1)$$

$$\mathrm{pK_{a1}} = \mathrm{pH} + \log\frac{[\mathrm{PDMPO}]}{[\mathrm{PDMPOH}^{+}]} \qquad (2)$$

Adopting $\mathrm{E_{1g}} = \mathrm{Exp}[\mathrm{pK_{a1}} - \mathrm{pH}]$ which is a function of pH and model variable $\mathrm{pKa_1}$ we may rewrite:

$$\mathrm{E_{1g}} = \frac{[\mathrm{PDMPO}]}{[\mathrm{PDMPOH}^{+}]} \qquad (3)$$

Second protonation in the ground state:

$$\mathrm{PDMPOH}^{+} + \mathrm{H}^{+} = \mathrm{PDMPOH}_2^{2+} \qquad (4)$$

$$\mathrm{pK_{a2}} = \mathrm{pH} + \log\frac{[\mathrm{PDMPOH}^{+}]}{[\mathrm{PDMPOH}_2^{2+}]} \qquad (5)$$

Analogously to the introduction of Eq. (4), using $\mathrm{E_{2g}} = \mathrm{Exp}[\mathrm{pK_{a2}} - \mathrm{pH}]$ as a function of pH and a model variable $\mathrm{pK_{a2}}$, we may write:

$$\mathrm{E_{2g}} = \frac{[\mathrm{PDMPOH}^{+}]}{[\mathrm{PDMPOH}_2^{2+}]} \qquad (6)$$

At this point, we may introduce a material concentration constraint

$$[\mathrm{PDMPO}] + [\mathrm{PDMPOH}^{+}] + [\mathrm{PDMPOH}_2^{2+}] = 1 \qquad (7)$$

Since emission efficiency of each of the forms is about the same, a practical implication of the constraint is that we may normalize a spectrum detected at any specific pH on its integral intensity. Another implication is that we may rewrite Eq. 3 as

$$E_{1g} = \frac{1-[PDMPOH^+]-[PDMPOH_2^{2+}]}{[PDMPOH^+]} \qquad (8)$$

to express

$$[PDMPOH^+] = \frac{1-[PDMPOH_2^{2+}]}{E_{1g}+1} \qquad (9)$$

We use Eq.9 to update Eq. 6 as:

$$E_{2g} = \frac{1-[PDMPOH_2^{2+}]}{(E_1+1)[PDMPOH_2^{2+}]} \qquad (10)$$

and to express concentration of the double protonated chromophore in dependence on pH, and variables pK_{a1} and pK_{a2}:

$$[PDMPOH_2^{2+}] = \frac{1}{E_{1g}E_{2g}+E_{2g}+1} \qquad (11)$$

Next, combining Eq. 9 and 11, we may derive concentration of the single protonated chromophore in dependence on pH, and variables pK_{a1} and pK_{a2}:

$$[PDMPOH^+] = \frac{E_{2g}}{E_{1g}E_{2g}+E_{2g}+1} \qquad (12)$$

Finally, here, accounting the concentration constraint we may define concentration of the deprotonated chromophore in dependence on pH, and variables pK_{a1} and pK_{a2}

$$[PDMPO] = 1 - [PDMPOH^+] - [PDMPOH_2^{2+}] = \frac{E_{1g}E_{2g}}{E_{1g}E_{2g}+E_{2g}+1} \qquad (13)$$

While addressing equations 11, 12 and 13, we may consider pK_{a1} and pK_{a2} values as already discussed in literature. Specifically, previously we addressed pK_{a1} as experimentally identified at 4.87; and pK_{a2} (of deprotonation) as predicted at 6.78 [4]. In the following, we will review and alter the latter value.

Additionally, since slope of a titration curve may be slightly different while passing through the equality point, we may flex modelling the observed slopes introducing a weakly varying k_1 and k_2

$$E_{1g} = \mathrm{Exp}[k_1(pK_{a1} - pH)] \qquad (14)$$
$$E_{2g} = \mathrm{Exp}[k_2(pK_{a2} - pH)]$$

Accordingly, in electronic ground state, concentrations of $PDMPOH^+$ and $PDMPOH_2^{2+}$ chromophore in dependence on pH are:

$$[PDMPO] = \frac{E_{1g}E_{2g}}{E_{1g}E_{2g}+E_{2g}+1}$$
$$[PDMPOH^+] = \frac{E_{2g}}{E_{1g}E_{2g}+E_{2g}+1} \qquad (15)$$
$$[PDMPOH_2^{2+}] = \frac{1}{E_{1g}E_{2g}+E_{2g}+1}$$

2) Contributions of PDMPO protonation states into emission component accounting proton transfer in the electronic excited state in dependence on pH

Previously, co-existence of two emission bands specific to $PDMPOH^+$ and $PDMPOH_2^{2+}$ chromophores in broad range of pH value after passing pK_{a1} was escribed to electronic excited state proton transfer from water [4]. Subject of structural variance of water arrangement next to the chromophore, efficiency of such process is limited by life-time of the electronic excited state. Since not all $PDMPOH^+$ may under protonation in electronic excited state, we may introduce w parameter to describe the fractions. Accordingly, emission spectral signatures of PDMPO forms which do not experience proton transfer are:

$$[\mathrm{PDMPO}^*] = \mathrm{A}_0(1-\mathrm{w})\frac{\mathrm{E_{1g}E_{2g}}}{\mathrm{E_{1g}E_{2g}+E_{2g}+1}}$$
$$[\mathrm{PDMPOH}^{+*}] = \mathrm{A}_H(1-\mathrm{w})\frac{\mathrm{E_{2g}}}{\mathrm{E_{1g}E_{2g}+E_{2g}+1}} \qquad (16)$$
$$\left[\mathrm{PDMPOH}_2^{2+*}\right] = \mathrm{A}_{2H}(1-\mathrm{w})\frac{1}{\mathrm{E_{1g}E_{2g}+E_{2g}+1}}$$

while emission spectral signatures of PDMPO forms which do experience proton transfer are:

$$[\mathrm{PDMPO}^*] = \mathrm{B}_0\mathrm{w}\frac{\mathrm{E_{1e}E_{2e}}}{\mathrm{E_{1e}E_{2e}+E_{2e}+1}}$$
$$[\mathrm{PDMPOH}^{+*}] = \mathrm{B}_H\,\mathrm{w}\frac{\mathrm{E_{2e}}}{\mathrm{E_{1e}E_{2e}+E_{2e}+1}} \qquad (17)$$
$$[\mathrm{PDMPOH}_2^{2+*}] = \mathrm{B}_{2H}\,\mathrm{w}\frac{1}{\mathrm{E_{1e}E_{2e}+E_{2e}+1}}$$

where

$$\mathrm{E_{1e}} = \mathrm{Exp}[\mathrm{k}_1(\mathrm{pK}_{a1}^* - \mathrm{pH})] \qquad (18)$$
$$\mathrm{E_{2e}} = \mathrm{Exp}[\mathrm{k}_2(\mathrm{pK}_{a2}^* - \mathrm{pH})]$$

2) Modelling of PDMPO emission in water

To model emission dependences on pH we have

1) A_0 (B_0), A_H (B_H), and A_{2H} (B_{2H}) factors to scale normalized emission spectra at pH = 14, 7 and 2.2, respectively as used to model properties of chromophores, which do not (or do) experience proton transfer in the excited state. In case oscillator strengths of considered transition is about the same (TDDFT theory suggests this), according to the material constraint by Eq. 7, we may consider $\mathrm{A}_0 = \mathrm{A}_H = \mathrm{A}_{2H} = 1$, as well as $\mathrm{B}_0 = \mathrm{B}_H = \mathrm{B}_{2H}$. Often, oscillator strength of transitions in excited state are different then such in the ground state;

2) $\mathrm{pK_{a1}}$ (pK_{a1}^*), and $\mathrm{pK_{a2}}$ (pK_{a2}^*) are the acid dissociation constants for the chromophore forms in the electronic ground (excited) state, respectively;

3) k_1 and k_2 rates to address slopes of titration curves at equality points;

4) w parameter to describe fractions of chromophores which do and do not experience proton transfer in the excited state.

First, concerning $\mathrm{pK_{a2}}$, the results of our current modelling (please, see Figure S10 and Figure 6B) indicate that the cross point for the emission spectra is approximately somewhere near 6.78, however,

this is a result of the overlap of the contributions of the two electronically different species: please note the contributions we factored in Figure S10A and S10B. Global fitting suggests $\mathrm{pK_{a2}} = 13.7$.

Next, slopes of the titration curves may be according to initial values of $\mathrm{k_1} = \mathrm{k_2} = 1$. Noticing that emission $PDMPOH_2^{2+*}$ starts to diminish after pH = 12.5, we may set introductory $\mathrm{pK}^*_{a1} = 13$ and pK^*_{a2}=14. Ideally, fitting would require w parameter: for example, 0.5. Then, value of A_H is fixed to confirm the level of $PDMPOH^+$ emission at 442nm in the pH range from 6 to 10: see Figure S11C. Analogously, we fix B_{2H} to confirm the level of $PDMPOH_2^{2+*}$ emission at 538 nm in the pH range from 6 to 10: see Figure S11D. According to the defined B_{2H}, we suggest A_{2H} to reproduce best the experimental spectra in the pH range from 2 to 4. At this point, since $\mathrm{A_0}$ and $\mathrm{B_0}$ scale the same spectral component (green line in Figure S11A), we may set $\mathrm{A_0} = \mathrm{B_0}$ to reproduce the experimental spectrum at pH=14. Next, we may attune B_H to account spectral variance in the pH range from 12 to 14. Having such set, we may return to $\mathrm{k_1}$, $\mathrm{k_2}$ and pKa values to improve the titration slopes.

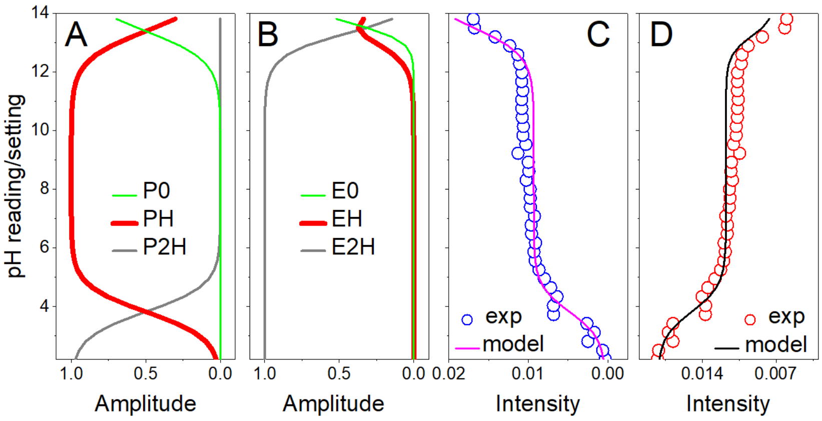

Figure S11. *pH components of PDMPO emission model in dependence on pH while in water.* ***A****: fitted to experimental data expressions of Eq. 16 to present pH dependences of* $PDMPOH_2^{2+}$*,* $PDMPOH^+$*and PDMPO (grey, red and green line, respectively), when* $A_0 = A_H = A_{2H} = 1$ *and* $w = 0$*.* ***B****: fitted to experimental data expressions of Eq. 17 to present pH dependences of* $PDMPOH_2^{2+*}$*,* $PDMPOH^{+*}$*, and* $PDMPO^*$ *(grey, red and green line, respectively), when* $\mathrm{B_0} = \mathrm{B}_H = \mathrm{B}_{2H} = 1$ *and* $\mathrm{w} = 1$*.* ***C*** *and* ***D****: comparison of experimentally detected pH dependence of emission at 442 and 538 nm (blue and red circle lines) with the correspondent dependences by the model (magenta and black lines).*

In this procedure, ratio between PDMPO chromophores to experience proton transfer in the electronic excited state or not, w, is one strong variable, while all others are to attune while monitoring spectral (Figure S12B-S12D) and pH (Figure S11C-S11D) selected data slices. In Table S3, we present a set of parameters to match modeled properties with the experimental ones for PDMPO in water, as we show in Figures S10-S12. Contributions of the considered spectral components are identical to satisfy the material constraint by Eq. 7.

Table S3. *Fitted parameters to model electronic properties of PDMPO in water in dependence on pH.*

$\mathrm{pK_{a1}}$	$\mathrm{pK_{a2}}$	$\mathrm{k_1}$	$\mathrm{k_2}$	$\mathrm{A_0}$	A_H	A_{2H}	w
4.2	13.8	2.2	2	1.0	1.0	1.0	0.45
pK^*_{a1}	pK^*_{a2}			$\mathrm{B_0}$	B_H	B_{2H}	$1 - \mathrm{w}$
13.8	14			1.0	1.0	1.0	0.55

Here, it is important to stress, that the emission experiment employs relatively weak continuous wave radiation: at any time, ratio of excited molecules to not excited molecules is very small. It means that, even though to discuss the detected emission spectrum we have to account contribution of proton transfer in the electronic excited states, Figure S10A describes pH dependent electronic properties of almost all molecules in the sample, which are not excited by light. As we will address later, this, however, does not undermine the considered sensorial capacity of PDMPO chromophore as a silicaphilic probe.

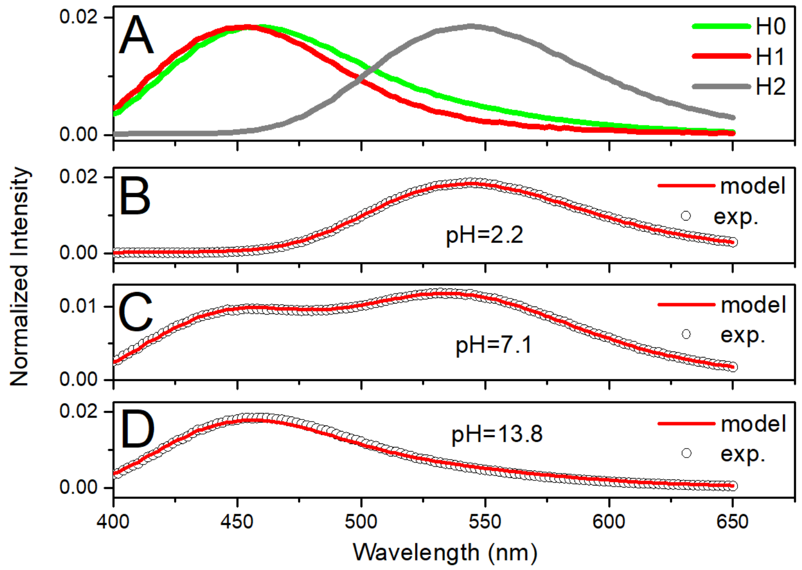

***Figure S12**. Spectral components of PDMPO emission model in dependence on pH while in water. **A**: emission spectra of $PDMPOH_2^{2+}$, $PDMPOH^+$ and PDMPO (grey, green, and red line, respectively), which we used for modelling emission dependence on pH as shown in Figure S10. Emission spectra of $PDMPOH_2^{2+}$ and PDMPO are experimental measurements under pH = 2.2 and 13.8. We receive emission spectrum of $PDMPOH^+$ subtracting scaled spectrum of $PDMPOH_2^{2+}$ from experimental data measured at intermediate pH: for example, pH = 9.5. **B**, **C**, and **D**: comparison of modelled spectra (red lines), which are horizontal slices from the model sum 2D data as shown in Figure S10, with the correspondent horizontal slices (open circles lines) from the experimental 2D data as shown in Figure S10 in dependence on pH as indicated.*

2) Direct application of the model for PDMPO emission in water when silica nanoparticles are present

Having modelling of PDMPO properties done when in water, we would like to apply the model without strong changes to see how this would describe experimental results in presence of silica nanoparticles. To start, however, we have to account one introductory peculiarity: experimental data (see Figure S13D) demonstrates a blue shift of the emission when pH changes from 3 to 3.6. We develop the model not to account any spectral diffusion. The spectral components before and after the shift can be factored and adopted in the model, but we have to explain such manipulations and why they were not necessary for PDMPO in water.

Considering the results of our TD-DFT efforts, the observation is possible to interpret: in this range of pH, $PDMPOH_2^{2+}$ molecules next to silica may experience the most acidic silanol moieties start to transfer proton to more basic ambient. While below pK_{a1}, theory predicts that emission of $PDMPOH_2^{2+}$ should shift blue when instead of neutral silica surface it may associate with a siloxide defect. Presence of such a defect introduces stronger interactions of $PDMPOH_2^{2+}$ electronics to admix

with silica components. In result, HOMO, LUMO and the next transitions shift into near infrared. Since they are of rather low oscillation strengths, they do not provide effective pathways to support emission, and, according to the theory outcome, the emission then happens from a higher energy electronic subset (not from LUMO): see Figure 4 of the main text. This we may take into account in our modelling. Specifically, in Figure S14A, we present a blue line spectrum, which in pH range from 4 to 10 should present electronic properties of $PDMPOH_2^{2+*}$next to silica with a siloxide defect. This double protonated form is present at the elevated pH due to the same excited state proton transfer from water, as we discussed already. Its emission maximum is blue shifted comparing to that of the grey line spectrum, which is specific to $PDMPOH_2^{2+*}$ next to neutral silica, as expected at pH=2. We receive blue line emission spectral signature of $PDMPOH_2^{2+*}$ subtracting scaled spectrum of $PDMPOH^+$ from experimental data measured at intermediate pH: for example, pH = 9.5.

Table S4. *Parameters used to address electronic properties of PDMPO in presence of silica nanoparticles in water and in dependence on pH.*

pK_{a1}	pK_{a2}	k_1	k_2	A_0	A_H	A_{2H}	w
3.8	13.9			1.0	1.0	1.0	0.45
pK^*_{a1}	pK^*_{a2}	2	1.7	B_0	B_H	B_{2H}	$1 - w$
13.9	14			1.0	1.0	1.0	0.55

Using the factored spectral components: see Figure S14A, we apply the model developed for PDMPO in water under parameters listed in Table S4 to present result in Figures S13-S15.

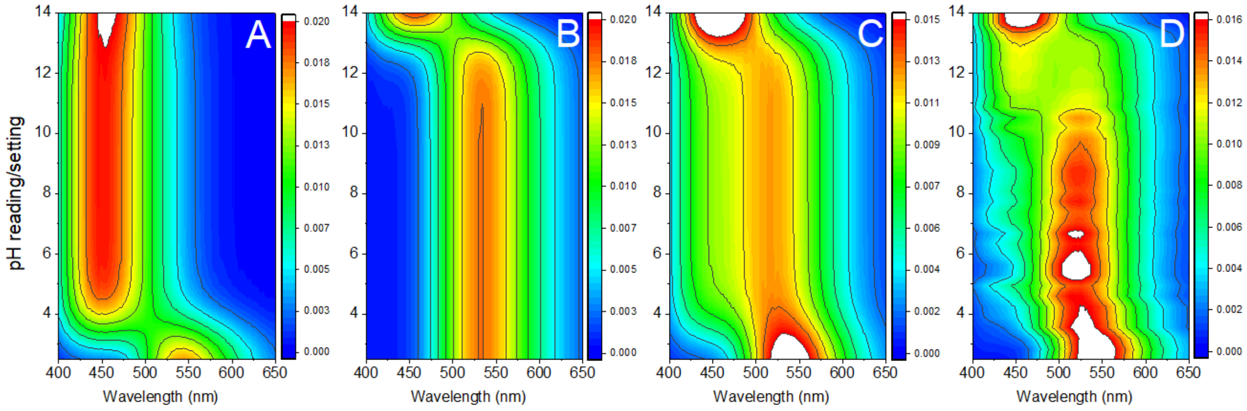

Figure S13. *Application of the "aqueous" model to address emission properties of PDMPO in presence of silica nanoparticles in water.* ***A****: computed emission of PDMPO which does not experience proton transfer in electronic excited state.* ***B****: computed emission of PDMPO which does experience proton transfer in electronic excited state.* ***C****: computed total emission.* ***D****: experimental data where each spectrum is normalized on its sum intensity.*

Direct application of the "aqueous" model indicates reproduction of spectral properties at very low pH = 2.5, and in the pH range from 10.5 to 14: see Figures S14B, S14C, S14E, S15C and S15D. It is interesting and important that in the pH range from 10.5 to 12, the ratio of the emission bands resembles that for PDMPO in water as shown in Figure S12C, which is conserved in the pH range from 5 to 12 as observed in the experiment on PDMPO in water: see Figures S11D, S11E. In contrast, application of the "aqueous model" to the results of emission spectroscopy on PDMPO in presence of silica fails to reproduce the ratio of the two emission bands (Figure S14D) as conserved for such system in the pH range from 3.8 to 10: see Figures S15C and S15D.

The observed switch of the band ration on pH when next to silica plays the central role in sensorial capacity of PDMPO to silica surface local ionicity. The mechanism of this we may explain using results of DFT.

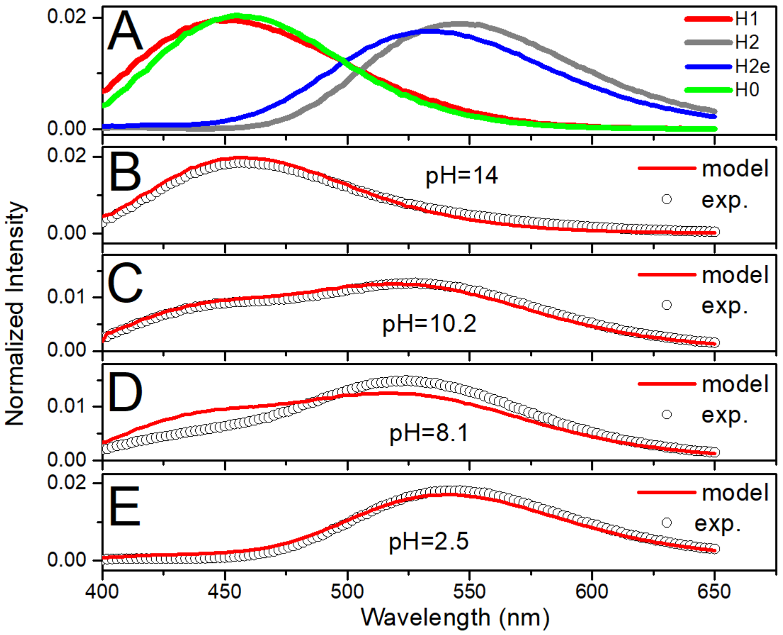

Figure S14. *Spectral components of PDMPO emission in presence of silica nanoparticles in water when applying "aqueous" model.* ***A***: *emission spectra of $PDMPOH_2^{2+}$ at neutral silica, $PDMPOH_2^{2+*}$ at silica with sparce siloxide defects, $PDMPOH^+$ at polar silica surface, and PDMPO at polar silica surface (grey, blue, green, and red line, respectively), which we used for modelling emission dependence on pH as shown in Figure S13. Emission spectra of $PDMPOH_2^{2+}$ at neutral silica and PDMPO at silica defect(s) are experimental measurements under pH = 2.2 and 13.8. We receive emission spectrum of $PDMPOH^+$ at silica defect(s) subtracting scaled spectrum measured under pH=5 from experimental data measured under pH = 11. Emission spectral $PDMPOH_2^{2+*}$ at silica defect is experimental data measured at intermediate pH minus scaled spectrum of $PDMPOH^+$ at silica defect(s).* ***B-E***: *comparison of modelled spectra (red lines), which are horizontal slices from the model sum 2D data as shown in Figure S13, with the correspondent horizontal slices (open circles lines) from the experimental 2D data as shown in Figure S13 in dependence on pH as indicated.*

First of all, above $\mathrm{pK_{a1}}$, observation of the emission band at 530 nm is the signature that while in the excited state some of $PDMPOH_2^{2+*}$ may receive proton from water. Relative increase or decrease of such emission (comparing to the band at 460 nm) correspond to increase or decrease of the proton transfer probability, which is limited by the lifetime of the excited state and geometry of aqueous moieties proximal to the pyridine nitrogen.

Theory suggests that within the pH range from 2 to 3.8, the system accounts double protonated chromophores to interact (not too strongly) with oxygen of silanol moieties (of neutral silica surface) either directly or via water bridges. When above $\mathrm{pK_{a1}}$=3.8, interactions of $PDMPOH^{1+}$ dimethyleamine group with relatively sparce siloxide moieties are energetically favorable. At the same time, the deprotonated pyridine may interact with water, with a silanol groups either directly or via water bridging. Since not ionic, such interactions are not strong and dynamic to open a large and flexible configuration space for such pyridine moiety. When photo-excited under the geometry of water bridging to hydrated silanol, we should expect very effective proton transfer, due to both, geometry of water bridging and neutrality of dominating silanol moieties. Such regime may provide the

dominance of the emission at 530 nm as conserved in the pH range from 3.8 to 10: see Figures S14D, S15C and S15D.

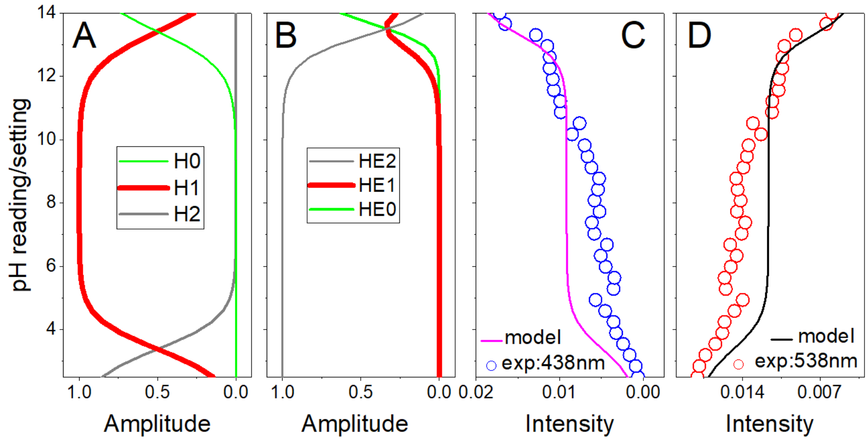

***Figure S15**. pH components of PDMPO emission model in dependence on pH while in water when applying "aqueous" model. **A**: fitted to experimental data expressions of Eq. 16 to present pH dependences of $PDMPOH_2^{2+}$, $PDMPOH^+$ and PDMPO (grey, red and green line, respectively), when $A_0 = A_H = A_{2H} = 1$ and $w = 0$. **B**: fitted to experimental data expressions of Eq. 17 to present pH dependences of $PDMPOH_2^{2+*}$, $PDMPOH^{+*}$ and $PDMPO^*$ (grey, red and green line, respectively), when $\mathrm{B}_0 = \mathrm{B}_H = \mathrm{B}_{2H} = 1$ and $\mathrm{w} = 1$. **C** and **D**: comparison of experimentally detected pH dependence of emission at 442 and 538 nm (blue and red circle lines) with the correspondent dependences by the model (magenta and black lines).*

Polarity of silica surface arise from the proton dissociation of surface acidic silanol groups and the pK_a reported for this reaction ranges from 4 to 7.5 (most frequently around 6–7) [8-11]. Additionally, there are experimental observations to indicate that there are two types of silanol groups: with silica pK_a values around 4.5–5.5 (15–19% of the total) and 8.5–9.9 (81–85%) [12,13] and theoretical studies to question the nature of possibly relevant structural states [14]. It is obvious that silica nanoparticles we used in our experiment demonstrated the second deprotonation event with its averaged equidistant point in the pH range between 8 and 10. This is where the ratio of the emission bands is changing. If using confocal microscopy, we there is a half-wavelength (200-300 nm) spatial limit to distinguish spatial diversity of ionic state, which can be correlated with chemistry of biosilica [15].

At this point, it is important to relate theory predictions for electronic responses of single protonated $PDMPOH^+$ when it faces silica surface to become polar after passing its second deprotonation. When a sufficient surface density of siloxides, beside polar binding of dimethyleamine group to a silica anionic site, deprotonated pyridine moiety may water bridge with soloxide as well: there is a significant energy benefit. However, under such geometry, the very proximity of the negatively charged siloxide should impose an electric force to prevent proton transfer via bridging water and from any neighbor aqueous cluster to the nitrogen of the pyridine moiety. Proton transfer may require a favorable relative reorientation to search a suitable aqueous donor. This is analogous to the situation of $PDMPOH^{+*}$ in water. When higher polarity of silica surface, the increasing population of single protonated chromophores, where pyridine group associates with a siloxide, is to reduce the number of excited $PDMPOH^+$ susceptible to except proton from nearby water. This is what determine the relative increase of the emission of the band at 430 nm when pH value becomes larger than 8 to stimulate the second deprotonation of the used silica nanoparticles.

When using microscopy, there is a chance to distinguish variance of local pK_a under special discretion down to 200 nm. This is particularly attractive to understand dynamics of material exchange with environment for organisms with silica skeletal elements to sustain healthy biochemistry. This concerns engineering of mineral implants to support healthy physiology on microscale.

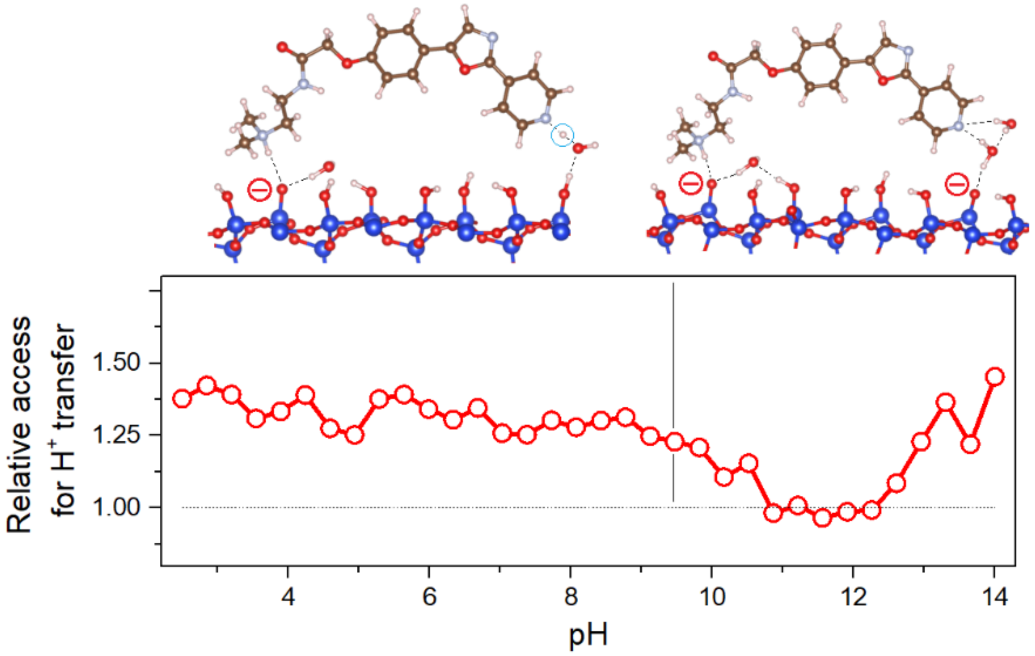

***Figure S16**. pH dependent w-function: half of it provides a pH-modulation for w factor in Eq. 16 and 17 to amend application of "aqueous" model in description of emission properties of PDMPO in presence of silica in water. w-function is larger or equal 1, whenever probability of $PDMPOH^{+*}$ to accept proton is larger or comparable to that when PDMPO is in water.*

Having the suggested explanation, we may adopt the difference of the simulated and observed pH dependence, as we plot in Figures S15C and S15D, to compute w-function, where the weights of $PDMPOH^{+}$ chromophores to undergo proton transfer from water in excited state or not is in dependence on pH and according to deprotonation process of the selected silica substrate: see Figure S16.

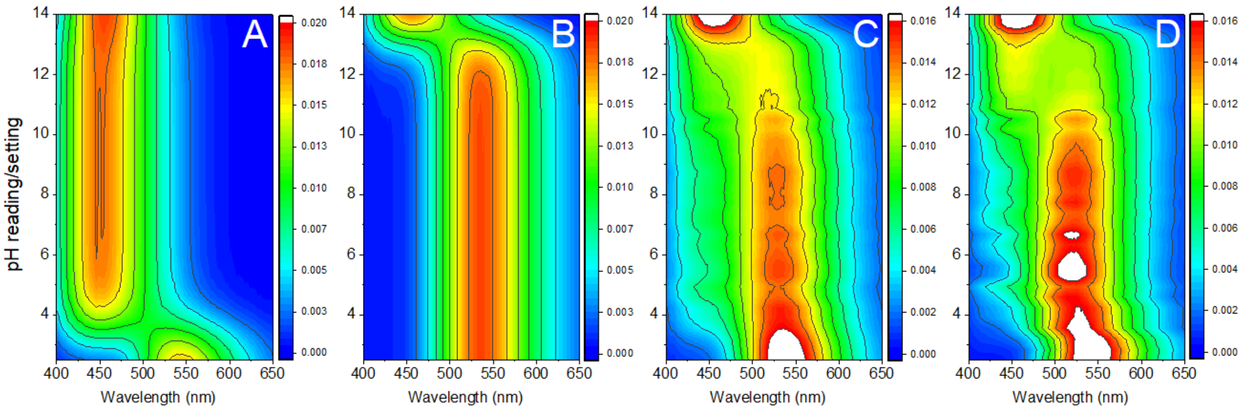

***Figure S17.** Application of w-function amended the "aqueous" model to address emission properties of PDMPO in presence of silica nanoparticles in water. **A**: computed emission of PDMPO which does not experience proton transfer in electronic excited state. **B**: computed emission of PDMPO which does experience proton transfer in electronic excited state. **C**: computed total emission. **D**: experimental data where each spectrum is normalized on its sum intensity.*

In Figures S17-S19, we present results of application of the “aqueous” model amended by w-function to address emission properties of PDMPO in presence of silica nanoparticles in water.

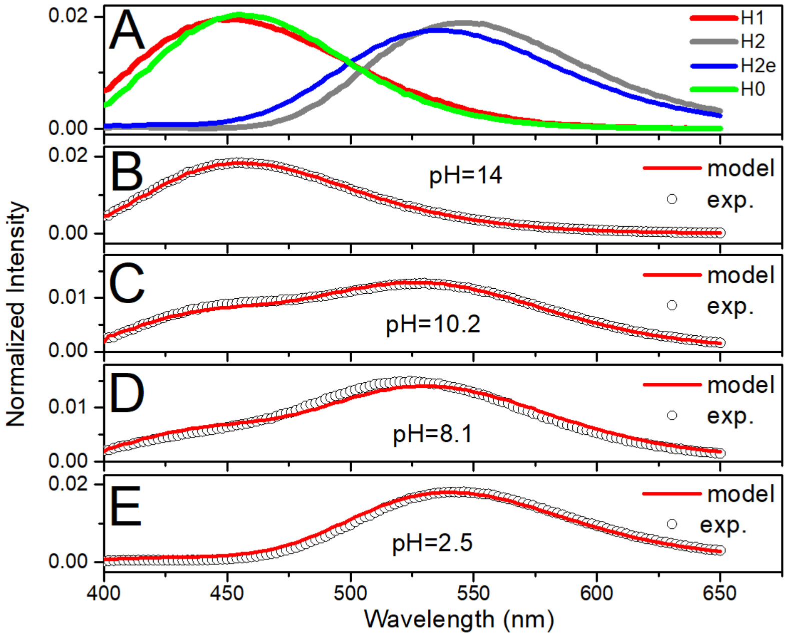

***Figure S18**. Spectral components of PDMPO emission in presence of silica nanoparticles in water when applying “aqueous” model under w-function pH-dependent correction. **A**: emission spectra of $PDMPOH_2^{2+}$ at neutral silica, $PDMPOH_2^{2+*}$ at silica with sparce siloxide defects, $PDMPOH^+$ at polar silica surface, and PDMPO at polar silica surface (grey, blue, green, and red line, respectively), which we used for modelling emission dependence on pH as shown in Figure S17. Emission spectra of $PDMPOH_2^{2+}$ at neutral silica and PDMPO at silica defect(s) are experimental measurements under pH = 2.2 and 13.8. We receive emission spectrum of $PDMPOH^+$ at silica defect(s) subtracting scaled spectrum measured under pH=5 from experimental data measured under pH = 11. Emission spectral $PDMPOH_2^{2+*}$ at silica defect is experimental data measured at intermediate pH minus scaled spectrum of $PDMPOH^+$ at silica defect(s). **B**-**E**: comparison of modelled spectra (red lines), which are horizontal slices from the model sum 2D data as shown in Figure S17, with the correspondent horizontal slices (open circles lines) from the experimental 2D data as shown in Figure S17 in dependence on pH as indicated.*

The accessibility w-function reflects consequences of hydrogen bond dynamics according to the nature of silica surface defects and nature of the silicaphilic PDMPO chromophore to compete for association with the surface defects. This we discuss in the main text.

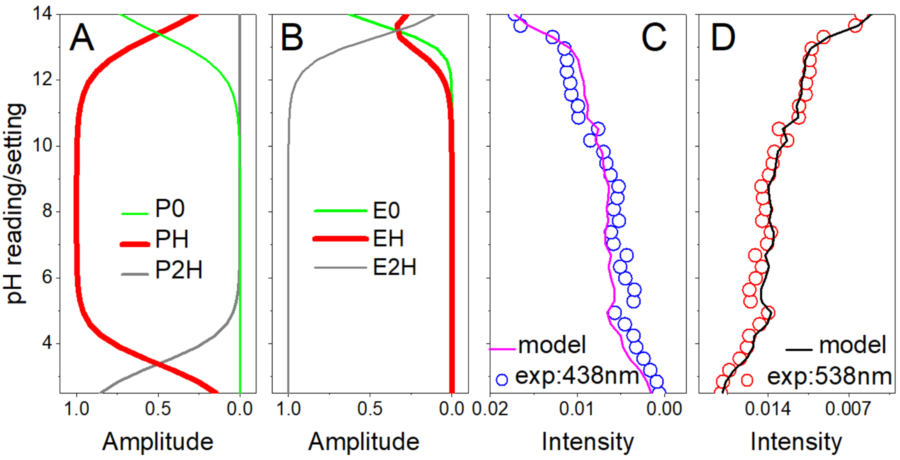

***Figure S19**. pH components of PDMPO emission model in dependence on pH while in water when applying "aqueous" model under w-function pH-dependent correction. **A**: fitted to experimental data expressions of Eq. 16 to present pH dependences of PDMPOH_2^{2+}, PDMPOH^+ and PDMPO (grey, red and green line, respectively), when $A_0 = A_H = A_{2H} = 1$ and $w = 0$. **B**: fitted to experimental data expressions of Eq. 17 to present pH dependences of PDMPOH_2^{2+*}, PDMPOH^{+*} and PDMPO* (grey, red and green line, respectively), when $\mathrm{B}_0 = \mathrm{B}_H = \mathrm{B}_{2H} = 1$ and $\mathrm{w} = 1$. **C** and **D**: comparison of experimentally detected pH dependence of emission at 442 and 538 nm (blue and red circle lines) with the correspondent dependences by the model (magenta and black lines).*

REFERENCES

1. T. L. Bahers, C. Adamo, I. Ciofini. A Qualitative Index of Spatial Extent in Charge-Transfer Excitations. J. Chem. Theor. Comp. 2011, 7, 2498-2506.
2. Becke, A.D. Density-functional exchange-energy approximation with correct asymptotic behavior. *Phys. Rev. A* **1988**, 38, 3098-3100.
3. M. J. Frisch, G. W. Trucks, H. B. Schlegel et al. Gaussian 16, Revision A.03. Gaussian, Inc., Wallingford CT, 2016.
4. M. Parambath, Q. S. Hanley, F. J. Martin-Martinez, T. Giesa, M. J. Buehler and C. C. Perry, Phys. Chem. Chem. Phys., 2016, 18, 5938.
5. M. Elstner, D. Porezag, G. Jungnickel, J. Elsner, M. Haugk, Th. Frauenheim, S. Suhai, and G. Seifert, *Phys. Rev. B*, 1998, 58, 7260.
6. P. Dera, J. D. Lazarz, V. B. Prakapenka, M. Barkley and R. T. Downs, *Phys. Chem. Miner.*, 2011, 38, 517-529.
7. K. Kihara, *Eur. J. Mineral.*, 1990, 2, 63-77.
8. Sonnefeld J, Löbbus M, Vogelsberger W (2001) Determination of electric double layer parameters for spherical silica particles under application of the triple layer model using surface charge density data and results of electrokinetic sonic amplitude measurements. Colloids Surf A Physicochem Eng Asp 195:215–225.
9. Hiemstra T, De Wit JC, Van Riemsdijk W (1989) Multisite proton adsorption modeling at the solid/solution interface of (hydr)oxides: a new approach. J Colloid Interface Sci 133:105–117.
10. Sverjensky DA (2005) Prediction of surface charge on oxides in salt solutions: revisions for 1:1 (M+L-) electrolytes. Geochim Cosmochim Acta 69:225–257.
11. Leroy P, Devau N, Revil A, Bizi M (2013) Influence of surface conductivity on the apparent zeta potential of amorphous silica nanoparticles. J Colloid Interface Sci 410:81–93
12. Allen LH, Matijevíc E, Meites L (1971) Exchange of Na+ for the silanolic protons of silica. J Inorg Nucl Chem 33:1293–1299;
13. Ong S, Zhao X, Eisenthal KB (1992) Polarization of water molecules at a charged interface: second harmonic studies of the silica/water interface. Chem Phys Lett 191:327–335.
14. Pfeiffer-Laplaud M, Costa D, Tielens F et al. (2015) Bimodal acidity at the amorphous silica/water interface. J Phys Chem C 119:27354–27362.
15. M. Parambath, A. Fayyaz, I. Efimov, Q. S. Hanley and C. C. Perry, *Analyst*, 2022, 147, 5586
